# Development of a four-gene prognostic model for pancreatic cancer based on transcriptome dysregulation

**DOI:** 10.18632/aging.102844

**Published:** 2020-02-20

**Authors:** Jie Yan, Liangcai Wu, Congwei Jia, Shuangni Yu, Zhaohui Lu, Yueping Sun, Jie Chen

**Affiliations:** 1 Department of Pathology, Peking Union Medical College Hospital, Chinese Academy of Medical Sciences and Peking Union Medical College, Beijing 100730, China; 2 Department of Obstetrics and Gynecology, Obstetrics and Gynecology Hospital of Fudan University, Shanghai 200011, China; 3 Institute of Medical Information, Chinese Academy of Medical Sciences and Peking Union Medical College, Beijing 100020, China

**Keywords:** prognostic prediction model, pancreatic cancer, TCGA, robust rank aggregation, WGCNA

## Abstract

We systematically developed a prognostic model for pancreatic cancer that was compatible across different transcriptomic platforms and patient cohorts. After performing quality control measures, we used seven microarray datasets and two RNA sequencing datasets to identify consistently dysregulated genes in pancreatic cancer patients. Weighted gene co-expression network analysis was performed to explore the associations between gene expression patterns and clinical features. The least absolute shrinkage and selection operator (LASSO) and Cox regression were used to construct a prognostic model. We tested the predictive power of the model by determining the area under the curve of the risk score for time-dependent survival. Most of the differentially expressed genes in pancreatic cancer were enriched in functions pertaining to the tumor immune microenvironment. The transcriptome profiles were found to be associated with overall survival, and four genes were identified as independent prognostic factors. A prognostic risk score was then proposed, which displayed moderate accuracy in the training and self-validation cohorts. Furthermore, patients in two independent microarray cohorts were successfully stratified into high- and low-risk prognostic groups. Thus, we constructed a reliable prognostic model for pancreatic cancer, which should be beneficial for clinical therapeutic decision-making.

## INTRODUCTION

Risk stratification (also commonly called “prognostic modeling”) is a useful tool in cancer management, since it enables timely interventions for high-risk patients while obviating unnecessary treatments for low-risk patients [1, 2]. Classical prognostic factors such as the clinical tumor-node-metastasis (cTNM) stage and pathological stage (pTNM) are not completely prognostically relevant in some patients [3–5]. Accordingly, the guidelines for prognostic assessments have been continually modified to improve their accuracy while reducing their complexity for daily clinical use [6–9].

Novel molecular factors such as immunoscores assessing *in situ* immune cell infiltration in tumors, abnormal DNA levels and mRNA levels are more accurate risk predictors than the existing tumor parameters [10–12]. High-throughput technologies provide an efficient means of measuring the molecular disruptions in tumors [13]. For example, a prognostic landscape of cancer was recently developed, which integrated the transcriptomes and clinical data of approximately 26,000 patients across 39 malignancies to establish the patterns and determinants of responses to targeted therapy [14]. Since numerous cancer-related microarrays and sequencing platforms have been generated in recent years, it is essential to integrate the large amounts of available data and translate these molecular findings into clinical decision-making tools. To this end, clinical data from The Cancer Genome Atlas (TCGA) Pan-Cancer analysis project have been integrated [15], and genotype-to-phenotype databases have been developed [16] for clinical interpretation [17].

Pancreatic cancer has a dismal prognosis, with a five-year survival rate of only 9% [18]. It is characterized by desmoplastic stroma, perineural invasion [5], invasiveness and immune suppression [13], which are largely responsible for the early metastasis [19], chemoresistance [20] and cachexia [21] observed in patients. Based on the transcriptome data of pancreatic cancer cells, tumors can be classified into the squamous, pancreatic progenitor, aberrantly differential endocrine exocrine, and immunogenic subtypes [13]. The squamous subtype is associated with a poor prognosis, and the immunogenic subtype involves the upregulation of gene networks for acquired immune suppression. A better understanding of the molecular landscape of pancreatic cancer would enable the development of novel therapeutic strategies to improve clinical outcomes and facilitate the stratification of patients into prognostic groups to guide personalized treatment. However, a comprehensive prognostic model with compatibility across different transcriptomic platforms and patient cohorts has not been systematically developed.

To determine the prognostic significance of the pancreatic cancer transcriptome, we screened multiple RNA-Seq and microarray datasets for genes that were differentially expressed between normal and tumorous tissues, and identified genes that were significantly associated with overall survival. We then developed a prognostic risk score and successfully validated it in three independent pancreatic cancer cohorts. We thereby devised a prognostic model that can predict the post-surgical prognosis of pancreatic cancer patients with moderate accuracy.

## RESULTS

### Combined analyses of multiple pancreatic cancer microarray datasets

We searched the Gene Expression Omnibus (GEO) database for all the human tissue microarrays that included pancreatic cancer tissues and paired/unpaired normal pancreatic tissues. Then, we used Transcriptome Analysis Console software (Applied Biosystems, version 4.0.2) to evaluate the data for hybridization and labeling controls. Affy [22] was used to assess RNA degradation, and simpleAffy [23] was used to determine the 3’-to-5’ ratios of *β-actin* and *GAPDH* (Supplementary Figure 2). Two pancreatic ductal adenocarcinoma (PDAC) datasets (GSE22780 and GSE27890) were thus excluded, and seven datasets (GSE32676, GSE16515, GSE71989, GSE41368, GSE15471, GSE28735 and GSE62452) were selected for further analysis (Table 1).

**Table 1:** Enrolled PDAC cases from seven GEO datasets after quality control.

**Country**	**Organization name**	**Series**	**Platform**	**Normal**	**Tumor**	**Quality control**	**Publication**
USA	University of Los Angeles	GSE32676	GPL570	7	25	Passed	[77]
USA	Mayo Clinic	GSE16515	GPL570	16	36	Passed	[78]
USA	University of Florida	GSE71989	GPL570	8	13	Excluded one non-tumor sample	[79]
Romania	ICI	GSE15471	GPL570	35	36	Excluded one normal tissue	[81]
Italy	Sapienza University of Rome	GSE41368	GPL6244	6	6	Passed	[80]
USA	NCI/NIH	GSE28735	GPL6244	44	43	Excluded one normal and two tumor samples	[82, 83]
USA	National Cancer Institute	GSE62452	GPL6244	61	67	Excluded two tumor samples	[84]

ICI: National Institute for Research in Informatics

**Figure 1:**
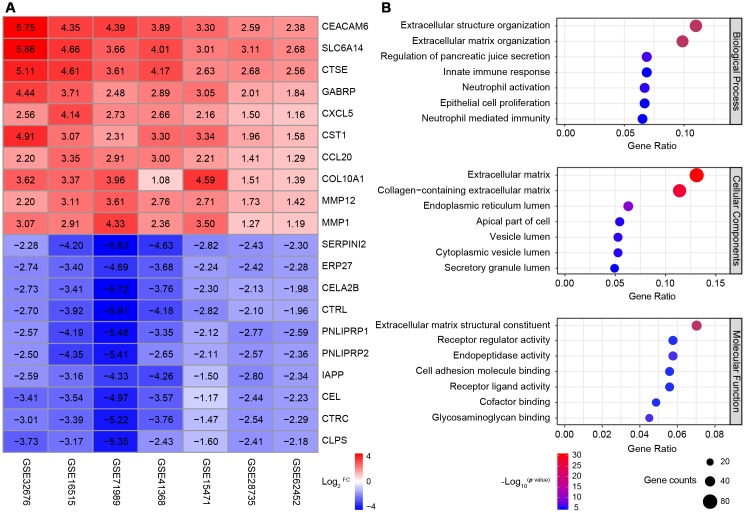
**Identification and GO analysis of DEGs in seven PDAC datasets.** (**A**) A heatmap of the top 10 significantly upregulated or downregulated genes. The expression of each gene in each dataset is shown in a colored box with the log_2_^FC^ inset. Red and blue boxes indicate upregulated and downregulated genes, respectively, and the color saturation correlates with the gene level. (**B**) GO analysis of all the DEGs. The seven most enriched GO terms in each category (BP, CC and MF) are listed next to the left axis. The size of the circle indicates the number of enriched genes, and the color is associated with the respective -log_10_^*p* value^.

After seven cases were excluded from these datasets, the data of 177 normal pancreatic tissue samples and 226 PDAC tissue samples were included in subsequent analyses. A robust rank aggregation analysis [24] identified 616 differentially expressed genes (DEGs) between the normal and PDAC samples across all datasets, with an adjusted *p* value < 0.05 and |log_2_^FC (fold change)^| > 1 as the cut-offs. Among these genes, 403 were upregulated and 213 were downregulated in PDAC tissues. The heatmap displaying the top 10 significantly overexpressed or suppressed genes is shown in Figure 1A.

Gene Ontology (GO) analysis of the DEGs revealed significant enrichment in the GO terms for 158 biological processes (BPs), 26 cellular components (CCs) and 28 molecular functions (MFs) (*p* value < 0.01 and *q* value < 0.01 as cut-offs) (Figure 1B). The top BP terms were related to three aspects: i) extracellular stroma formation, including extracellular structure organization and extracellular matrix organization, which was not surprising, since stiffness is the defining characteristic of PDAC; ii) immune cell responses, such as the innate immune response, neutrophil activation and neutrophil mediated immunity; and iii) fundamental pancreatic functions, such as regulating pancreatic juice secretion and epithelial cell proliferation. Major CC terms included the extracellular matrix and various components of the intracellular lumen and the apical part of the cell. The most enriched MF terms were extracellular matrix formation, cell adhesion and receptor ligand activity.

Thus, through a combined analysis of seven high-quality GEO microarrays, we identified 616 genes that were consistently differentially expressed between normal pancreatic tissues and PDAC tissues. Most of the DEGs were associated with the pancreatic extracellular stroma and the tumor immune microenvironment.

### Combined analyses of TCGA and GTEx RNA-Seq datasets

To determine whether the DEGs were independent of the detection method, we also analyzed their expression in PDAC RNA-Seq datasets. Since TCGA only contains data from four normal pancreatic tissue samples [25], we also included normal tissue data from the Genotype-Tissue Expression (GTEx) database, which contains normal tissue samples from 54 human body sites and is maintained by The Broad Institute of MIT and Harvard [26, 27]. The GTEx and TCGA RNA-Seq data and phenotypic information were obtained from the University of California Santa Cruz (UCSC) Xena platform (https://xena.ucsc.edu/), which is routinely updated and integrated [28].

**Figure 2:**
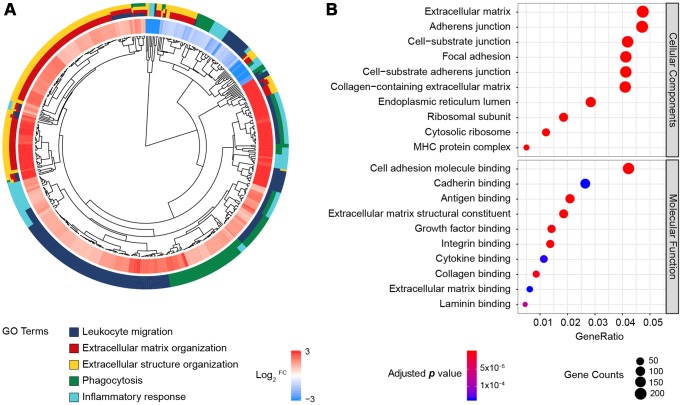
**GO enrichment analysis of the DEGs from the RNA-Seq datasets of TCGA and GTEx.** (**A**) GO Cluster. The inner dendrogram indicates the hierarchical clustering of the gene expression profiles, the outer circle represents the log_2_^FC^ of each DEG, with the color corresponding to the gene level, and the outermost circle represents the GO BP terms assigned to the gene. (**B**) The 10 most significantly enriched CC and MF terms. The size of a circle indicates the number of enriched genes, and its color corresponds to the adjusted *p* value.

In total, 171 normal and 178 pancreatic cancer samples were analyzed, and 5,886 DEGs were identified by the same cut-off criteria used to analyze the PDAC microarrays (|log_2_^FC^| > 1 and false discovery rate < 0.05). Among these genes, 2,980 were upregulated and 2,906 were downregulated in pancreatic cancer tissues relative to normal pancreatic tissues. These DEGs were enriched in 600 BP, 106 CC and 32 MF terms, and the most significantly enriched BP terms were related to leukocyte function, extracellular matrix formation, inflammation, etc. (Figure 2A). Consistent with the DEGs in the microarrays, most of the genes were enriched in the extracellular matrix or junctions for adhesion and antigen-binding functions (Figure 2B). Thus, both sequencing and multi-microarray data indicated that the tumor immune and stroma microenvironment was significantly disturbed during pancreatic tumorigenesis.

### Identification of 542 genes that were consistently differentially expressed across independent platforms

We next investigated whether the 616 DEGs identified by the seven microarrays were consistent with the 5,886 DEGs identified by PDAC transcriptome sequencing. When we compared the DEG profiles obtained by these two methods, we detected 542 common genes. Surprisingly, all of the overlapping genes displayed consistent expression trends in the two types of profiles (Supplementary Table 1).

Since transcription factors (TFs) and kinases are key components of cancer regulatory networks and are preferred targets for drug development [29, 30], we cross-referenced the DEGs with both the Cistrome Cancer human TF database [31] and a list of 518 human kinases [32]. Of the DEGs, 19 displayed TF activity (Table 2) and 16 were kinases, of which six belonged to the Tyrosine Kinase group (Table 3). Thus, we identified 542 pancreatic-cancer-related genes that were consistently dysregulated in both multi-microarray datasets and sequencing datasets, including 35 well-defined protagonists harboring core regulatory functions.

**Table 2:** The 19 differentially expressed TFs in pancreatic cancer.

**Gene**	**Seven GEO datasets**			**TCGA combined GTEx datasets**		
	**Log_2_^FC^**	***p* value**	**Adjusted *p* value**	**Log_2_^FC^**	***p* value**	**FDR**
*GCG*	-1.97	1.96E-11	4.90E-07	-1.13	0.038	0.039
*FOXQ1*	1.61	5.62E-09	1.41E-04	3.39	1.54E-51	7.57E-51
*DKK1*	2.29	1.65E-12	4.13E-08	2.47	1.36E-42	3.29E-42
*KLF5*	1.46	1.79E-07	4.49E-03	3.17	7.69E-48	2.49E-47
*AHR*	1.16	7.41E-08	1.85E-03	2.96	3.53E-56	1.30E-54
*ARNTL2*	1.61	5.71E-10	1.43E-05	1.94	2.39E-54	3.24E-53
*ID1*	1.25	9.58E-09	2.40E-04	2.15	3.63E-43	9.03E-43
*PPARG*	1.27	8.74E-08	2.19E-03	2.00	4.27E-47	1.31E-46
*LEF1*	1.45	4.60E-08	1.15E-03	1.80	2.38E-49	8.74E-49
*PTTG1*	1.13	4.99E-09	1.25E-04	2.06	1.29E-53	1.15E-52
*MXD1*	1.07	2.05E-08	5.13E-04	1.68	3.89E-51	1.79E-50
*DTL*	1.22	1.18E-10	2.94E-06	1.13	7.81E-53	5.14E-52
*ETV1*	1.04	9.97E-07	2.49E-02	1.29	7.00E-52	3.63E-51
*ZNF521*	1.19	1.99E-06	4.98E-02	1.05	1.29E-41	3.00E-41
*NCAPG*	1.01	1.14E-07	2.85E-03	1.07	8.97E-49	3.14E-48
*BCAT1*	-1.12	1.91E-08	4.78E-04	-1.51	1.91E-36	3.79E-36
*ZBTB16*	-1.51	6.22E-10	1.56E-05	-1.88	5.90E-43	1.45E-42
*NR5A2*	-2.12	3.54E-14	8.86E-10	-2.27	1.65E-48	5.66E-48
*CTRL*	-3.37	2.22E-19	5.55E-15	-8.38	8.32E-55	1.45E-53

**Table 3:** The 16 differentially expressed kinases in pancreatic cancer.

**Entrez ID**	**Gene**	**SK**	**Group**	**Family**	**Seven GEO datasets**			**TCGA combined GTEx datasets**		
					**Log_2_^FC^**	***p* value**	**Adjusted *p* value**	**Log_2_^FC^**	***p* value**	**FDR**
1969	*EPHA2*	SK122	TK	Eph	1.02	1.10E-06	0.028	2.77	1.21E-48	4.18E-48
4233	*MET*	SK227	TK	Met	1.17	1.07E-06	0.027	2.45	2.22E-49	8.18E-49
55359	*STYK1*	SK530	TK	TK-Unique	1.47	3.72E-09	9.30E-05	1.85	1.25E-53	1.13E-52
4486	*MST1R*	SK332	TK	Met	1.58	1.10E-07	0.003	1.66	4.74E-37	9.57E-37
9833	*MELK*	SK298	CAMK	CAMKL	1.54	3.07E-10	7.67E-06	1.57	2.93E-50	1.19E-49
983	*CDK1*	SK065	CMGC	CDK	1.03	1.99E-07	0.005	1.86	5.32E-52	2.82E-51
4751	*NEK2*	SK251	Other	NEK	1.08	1.05E-08	0.000	1.61	5.66E-53	3.91E-52
9448	*MAP4K4*	SK437	STE	STE20	1.01	2.85E-07	0.007	1.56	3.46E-53	2.55E-52
55872	*PBK*	SK529	Other	TOPK	1.10	3.76E-08	0.001	1.35	1.74E-53	1.47E-52
9891	*NUAK1*	SK195	CAMK	CAMKL	1.02	5.09E-08	0.001	1.38	3.78E-50	1.52E-49
701	*BUB1B*	SK053	Other	BUB	1.28	3.42E-09	8.56E-05	1.10	5.88E-49	2.09E-48
2043	*EPHA4*	SK124	TK	Eph	1.06	2.30E-07	0.006	1.27	5.14E-48	1.69E-47
4915	*NTRK2*	SK378	TK	Trk	-1.06	4.81E-07	0.012	-1.06	9.65E-30	1.65E-29
5166	*PDK4*	SK280	Atypical	PDHK	-1.76	6.11E-13	1.53E-08	-1.49	5.54E-19	7.88E-19
5063	*PAK3*	SK269	STE	STE20	-1.59	2.81E-12	7.02E-08	-2.09	8.26E-45	2.22E-44
8569	*MKNK1*	SK235	CAMK	MAPKAPK	-1.01	1.82E-08	0.000	-4.04	2.02E-55	4.75E-54

### The dysregulated transcriptome is associated with overall survival in pancreatic cancer patients

To determine the phenotypic relevance of the DEGs, we performed a weighted gene co-expression network analysis (WGCNA) [33] to identify gene modules (groups of highly interconnected genes) that were significantly associated with the clinico-pathological features of pancreatic cancer. Since sufficient sample sizes and adequate phenotypic data (including prognostic data) are prerequisites for analyzing gene co-expression networks that are associated with clinical characteristics, we only extracted the gene expression profiles and corresponding clinical data of the PDAC patients from TCGA who met these criteria (N = 135). Fourteen clusters (modules) of highly interconnected genes with co-expression similarity values > 0.75 for the module eigengenes were identified (Supplementary Figure 3, Supplementary Table 2).

**Figure 3:**
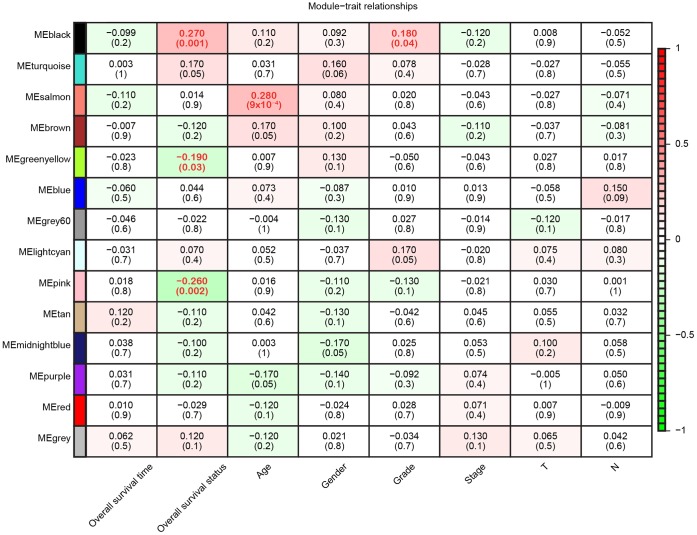
**Module-trait relationships.** Each row represents a color-coded module eigengene, each column represents a clinical trait, and each cell represents the Pearson correlation coefficient (top number) and *p* value (in parentheses) of the corresponding module-trait. The color of each cell indicates the degree of correlation, as shown in the key. Abbreviations: T, Primary tumor; N, Regional lymph nodes.

Since we had already identified the characteristic gene co-expression profiles of each module, we searched for significant correlations between the module eigengenes and clinical traits. Overall survival status was associated with three modules, although the Pearson correlations were weak; for example, the black module correlated positively (*r* = 0.27, *p* = 0.001) and the pink module correlated negatively with overall survival (*r* = -0.26, *p* = 0.002) (Figure 3). Additionally, age and tumor grade were associated with one module each, while the other four clinical traits we examined (gender, tumor stage, T classification and N classification) were not associated with any module. Thus, WGCNA analysis indicated that overall survival was the main clinical trait associated with the pancreatic cancer profiles in TCGA, so we focused on overall survival in our subsequent investigations.

### Construction of a prognostic model for pancreatic cancer

**Figure 4:**
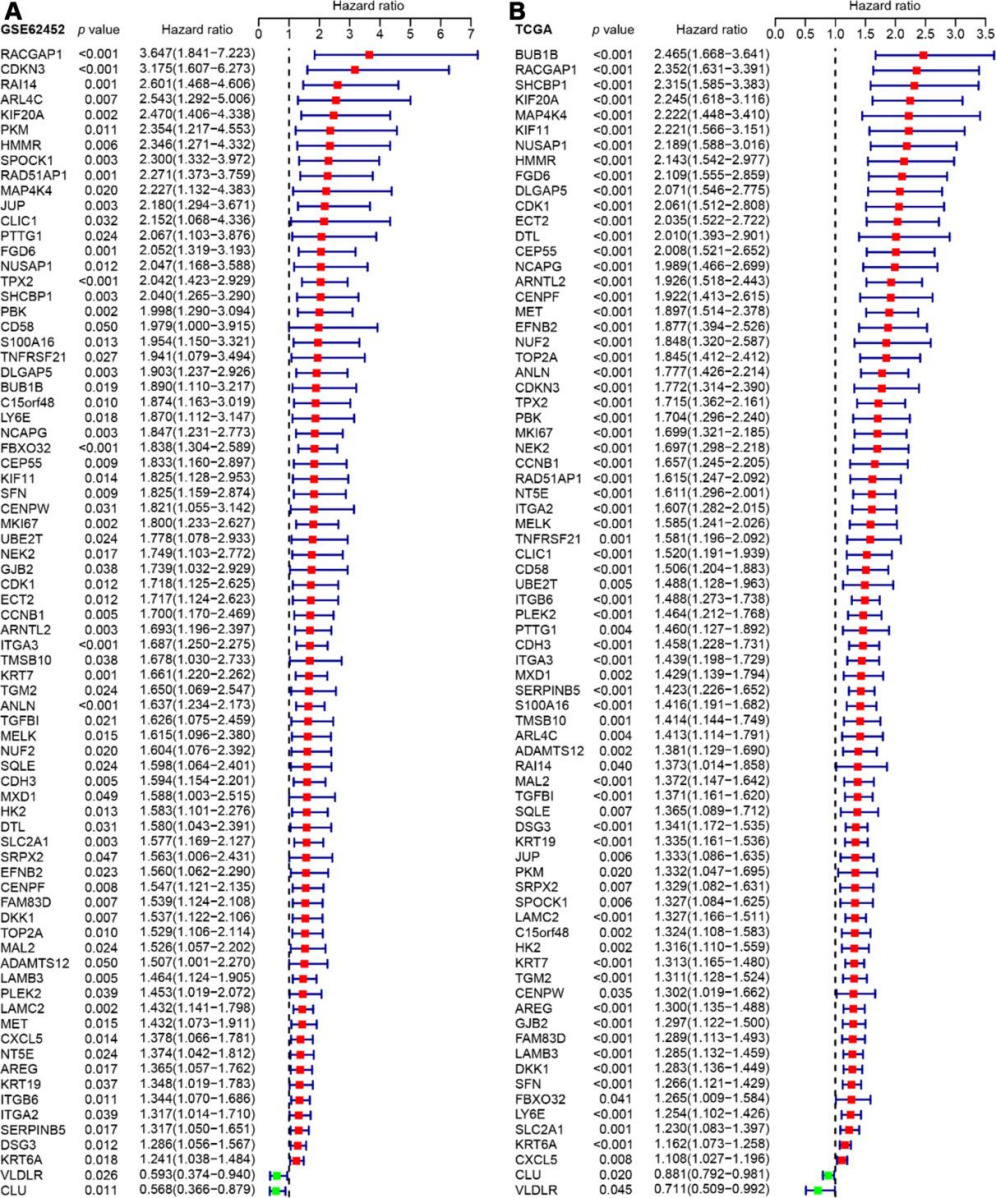
Forest plots visualizing the HRs of 76 prognostic DEGs identified by univariate Cox analysis of GSE62452 (**A**) and TCGA (**B**). The first three columns display the gene name, *p* value, and HR and 95% CI, respectively. In the forest plot, protective associations are shown in green and risk factors are shown in red.

Since GSE62452 and TCGA harbored an adequate number of cases and sufficient clinical follow-up data, we performed a univariate Cox regression analysis of these datasets to investigate the prognostic significance of the 542 consistently identified DEGs described above. A prognosis-associated gene was defined as impacting overall survival with a hazard ratio (HR) and 95% confidence interval (CI) greater than or less than 1. While 92 of the 542 DEGs had prognostic value in GSE62452 (*p* < 0.05), 76 of these 92 genes were still prognostically relevant in the cohort from TCGA (*p* < 0.05) (Figure 4).

**Figure 5:**
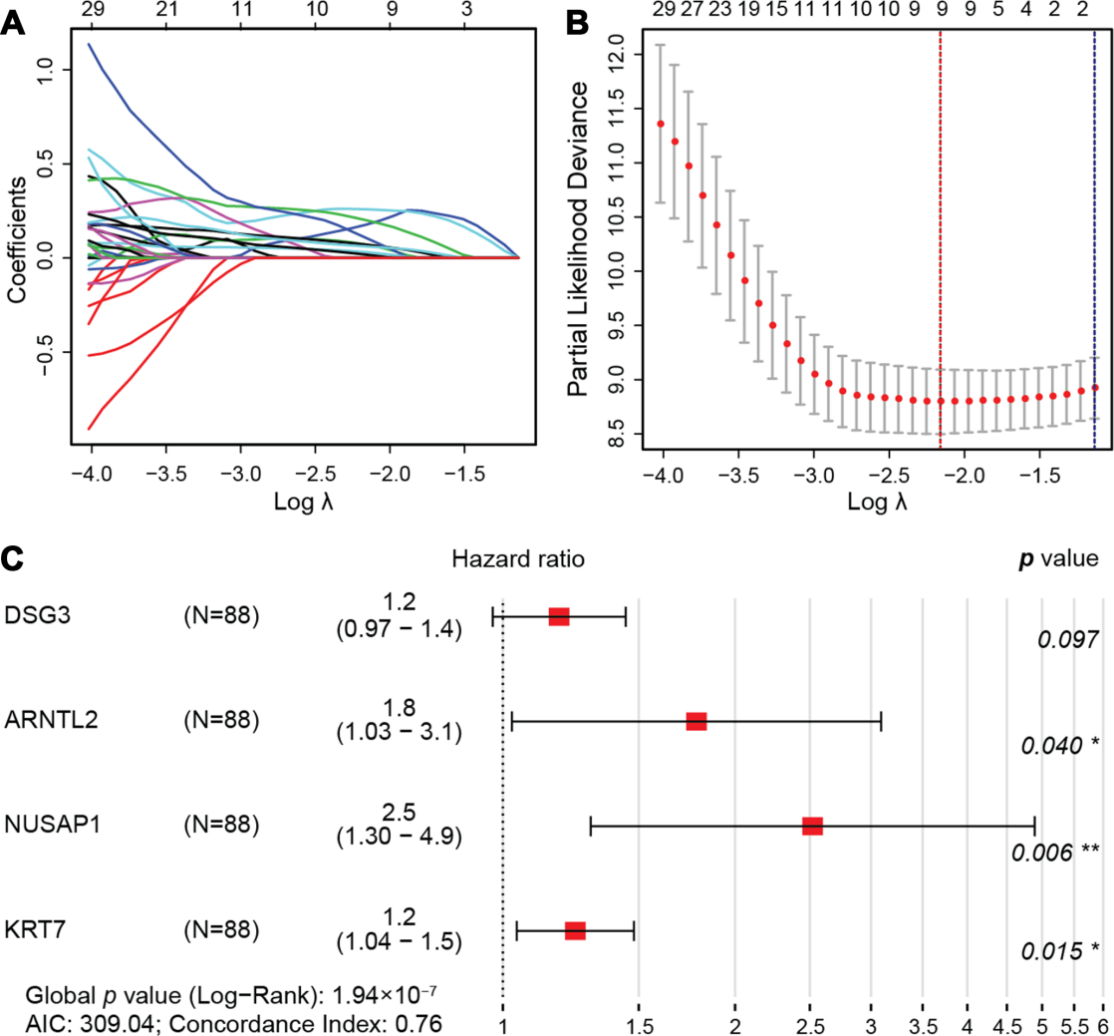
**LASSO regression model.** (**A**) LASSO coefficient profiles of the 76 prognostic DEGs. Each curve represents a coefficient, and the x-axis represents the regularization penalty parameter. As λ changes, a coefficient that becomes non-zero enters the LASSO regression model. (**B**) Cross-validation to select the optimal tuning parameter (λ). The red dotted vertical line crosses over the optimal log λ, which corresponds to the minimum value for multivariate Cox modeling. The two dotted lines represent one standard deviation from the minimum value. (**C**) HRs and 95% CIs of the four genes based on multivariate Cox regression analysis of the training cohort from TCGA.

A least absolute shrinkage and selection operator (LASSO) regression model [34] was then used to select key prognosis-associated genes. In the LASSO-penalized Cox regression, as log λ (a tuning parameter) changed, the corresponding coefficients of certain genes were reduced to zero, indicating that their effects on the model could be omitted because they were shrinking parameters (Figure 5A). Following cross-validation, nine genes achieved the minimum partial likelihood deviance (Figure 5B). Moreover, at this point, log λ was approximately -2.16, and the nine genes displayed non-zero effects, all contributing positive HRs to the model. The nine genes that were thus fit into the Cox model were *PBK*, which encodes MAPKK-like protein kinase; *DLGAP5*, which encodes a mitotic phosphoprotein; *RACGAP1*, also called Rac GTPase activating protein 1; *DSG3*, also called cadherin family member 6; *ARNTL2*, which functions as a TF; *NUSAP1*, also called nucleolar and spindle associated protein 1; *DKK1*, also called Dickkopf WNT signaling pathway inhibitor 1; *KRT7*, which encodes a cytokeratin; and *C15orf48*, a protein-coding gene that is also called chromosome 15 open reading frame 48.

Then, we randomly divided the 176 pancreatic cancer patients in TCGA (two cases in the enrolled TCGA pancreatic cancer cohort (N=178) were excluded due to insufficient follow-up data) into a training cohort (N = 88) for the construction of a prognostic model, and a validation cohort (N = 88) for internal self-validation (Table 4). Multivariate Cox regression analysis of the training cohort indicated that *ARNTL2*, *NUSAP1*, *DSG3* and *KRT7* were independent prognostic factors (Figure 5C). The prognostic risk score was calculated as: expression of *DSG3* × 0.17 + expression of *ARNTL2* × 0.58 + expression of *NUSAP1* × 0.92 + expression of *KRT7* × 0.22, where the numbers indicate the respective multivariate Cox regression coefficients.

**Table 4:** Clinico-pathological characteristics of patients in the training and self-validation cohorts of TCGA and two independent GEO validation datasets.

**Characteristics**	**TCGA training cohort**	**TCGA validation cohort**	**GSE62452 validation cohort**	**GSE28735 validation cohort**
	**(N = 88)**	**(N = 88)**	**(N = 64)**	**(N = 40)**
**Age at initial diagnosis (year)**	64.7 + 1.2	64.6 + 1.1	NA	NA
**Gender**			NA	NA
Male	53 (60.2%)	43 (48.9%)		
Female	35 (39.8%)	45 (51.1%)		
**Neoplasm histological grade**				NA
G1	13 (14.8%)	17 (19.3%)	2 (3.1%)	
G2	50 (56.8%)	44 (50.0%)	31 (48.4%)	
G3	22 (25.0%)	26 (29.5%)	29 (45.3%)	
G4	1 (1.1%)	1 (1.1%)	1 (1.6%)	
Not report	2 (2.3%)	0 (0.0%)	1 (1.6%)	
**Primary tumor (T)**			NA	NA
T1	3 (3.4%)	4 (4.5%)		
T2	13 (14.8%)	11 (12.5%)		
T3	71 (80.7%)	69 (78.4%)		
T4	1 (1.1%)	2 (2.3%)		
Not report	0 (0.0%)	2 (2.3%)		
**Tumor stage at diagnosis**				NA
Stage I	7 (8.0%)	14 (15.9%)	4 (6.3%)	
Stage II	76 (86.4%)	69 (78.4%)	45 (70.3%)	
Stage III	1 (1.1%)	2 (2.3%)	9 (14.1%)	
Stage IV	3 (3.4%)	1 (1.1%)	6 (9.4%)	
Not report	1 (1.1%)	2 (2.3%)	0 (0.0%)	
**Overall survival time (years)**	1.31 (0.79–1.83)	1.25 (0.67–1.95)	1.27 (0.74–2.27)	1.28 (0.6–2.03)
**Overall survival status**				
Alive	41 (46.6%)	43 (48.9%)	16 (25.0%)	13 (32.5%)
Dead	47 (53.4%)	45 (51.1%)	48 (75.0%)	27 (67.5%)

NA: Not available.

Thus, through univariate Cox regression analysis, LASSO model shrinkage and multivariate Cox model construction, we obtained four DEGs (*DSG3*, *ARNTL2*, *NUSAP1* and *KRT7*) for prognostic risk evaluation.

### Stratification of TCGA training and self-validation cohorts using the four-gene signature

**Figure 6:**
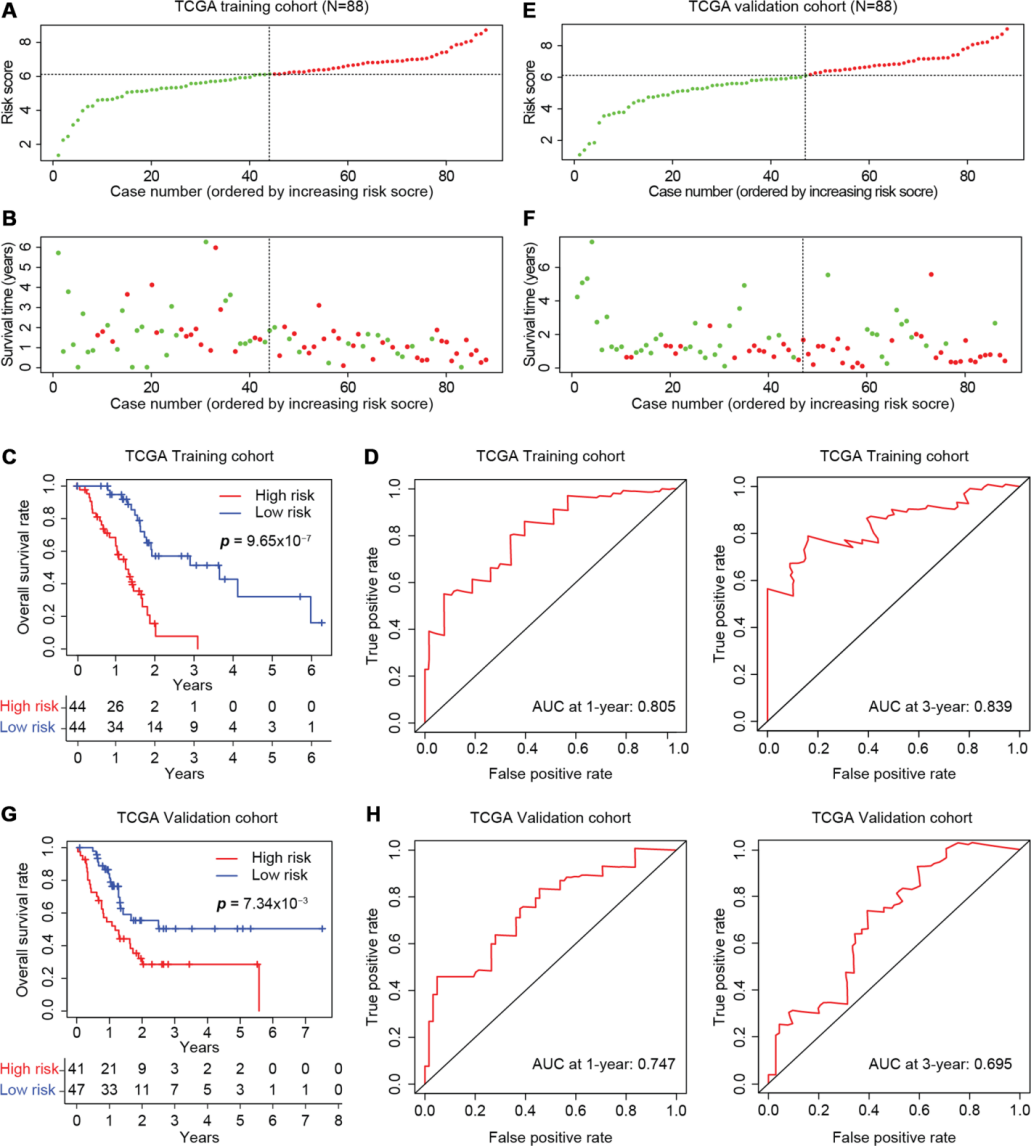
**Development of the prognostic scoring model in TCGA cohorts.** The distribution of risk scores is shown for the training (**A**) and validation (**E**) cohorts from TCGA. The dotted horizonal line indicates the cut-off level of the risk score used to stratify patients, and the dotted vertical line separates patients on the basis of low-risk (green) or high-risk (red). (**B**, **F**) The distribution of overall outcomes in the training (**B**) and validation (**F**) cohorts from TCGA. Surviving patients are shown in green, while deaths are shown in red. (**C**, **G**) Kaplan-Meier survival plots of patients predicted to be at risk for poor outcomes in the training (**C**) and validation (**G**) cohorts from TCGA. The number of patients remaining at a particular timepoint is shown at the bottom. (**D**, **H**) Time-dependent ROC curves for predicting one-year and three-year survival in the training (**D**) and validation (**H**) cohorts from TCGA.

We then divided the training cohort into high-risk and low-risk groups (Figure 6A). We used the median risk score (6.12) as the cut-off because the risk score usually exhibits a skewed distribution. The high-risk group displayed a higher frequency of poor survival outcomes than the low-risk group (Figure 6B). The three-year survival rates of the high-risk and low-risk groups were 7.78% and 51.3%, respectively (Figure 6C). To determine the predictive accuracy of this prognostic model, we performed a receiver operating characteristic (ROC) curve analysis, which demonstrated that the area under the curve (AUC) was 0.805 for one-year survival and 0.839 for three-year survival in the training cohort of TCGA (Figure 6D).

The model was further tested in the self-validation cohort by the same protocol (Figure 6E), which indicated that higher scores corresponded to worse overall survival (Figure 6F). The three-year survival rate was 28.6% in the high-risk group and 50.4% in the low-risk group (Figure 6G). Moreover, the AUCs for one-year and three-year survival in the validation cohort were 0.747 and 0.695, respectively (Figure 6H). Thus, the prognostic model successfully stratified pancreatic cancer patients from TCGA into high- and low-risk groups with moderate predictive power.

### The four-gene prognostic model is reliable in independent cohorts

**Figure 7:**
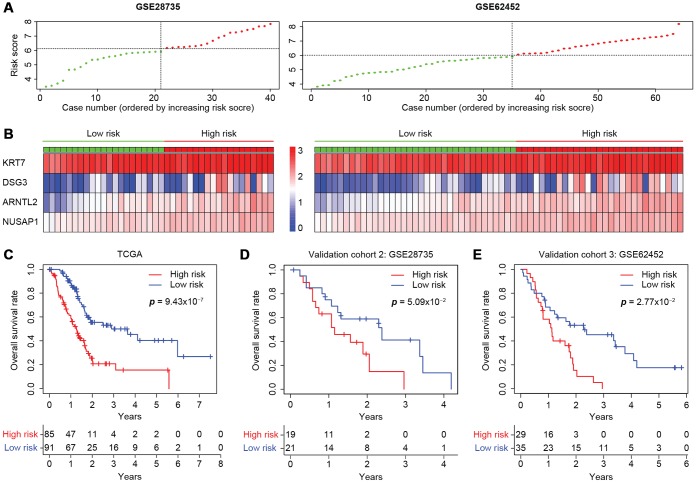
**Validation of the four-gene model in two independent microarray datasets.** (**A**) The risk score distribution in the GSE28735 and GSE62452 cohorts. (**B**) Heatmap displaying the levels of the four genes in the high- and low-risk groups. The color of each case corresponds to the log_2_^FC^ of the gene level, as shown in the key. (**C**–**E**) Kaplan-Meier survival plots of high- or low-risk patients in the cohorts from TCGA (**C**), GSE28735 (**D**) and GSE62452 (**E**). The number of patients remaining at a particular timepoint is shown at the bottom.

The predictive capacity of the four-gene model was further tested on two independent GEO microarray datasets (GSE28735 and GSE62452) that also included clinical data. The risk scores were calculated as described above (Figure 7A), and the expression of each gene was found to be greater in the high-risk group than in the low-risk group (Figure 7B). Consistent with the results in the entire TCGA cohort (Figure 7C), the risk score accurately stratified the patients of both datasets in terms of their survival outcomes. The three-year overall survival rates of the high- and low-risk groups in GSE28735 were 14.8% and 41.3%, respectively (Figure 7D), while those in GSE62452 were 5.15% and 45.3%, respectively, displaying an even greater prognostic difference (Figure 7E). Therefore, the four-gene signature is an accurate, reliable and independent predictive tool for determining the prognosis of pancreatic cancer patients.

## DISCUSSION

In this study, a pancreatic cancer prognostic model based on transcriptome dysregulation was developed, which was compatible across the microarray and RNA-Seq platforms and among different patient cohorts. Our prognostic model containing four DEGs (*DSG3*, *ARNTL2*, *NUSAP1* and *KRT7*) was used to determine the risk scores of pancreatic cancer patients, and patients with high risk scores were found to exhibit poor overall survival.

Molecular predictors such as the multi-gene expression assays in lung cancer [35] and renal cell carcinoma [36] or the immunoscore in colon cancer [10] have shown promise in facilitating clinical decision-making in large international validation studies. However, commonly mutated genes are not the primary determinants of PDAC prognosis [4]. On the other hand, transcriptome profiles have been successfully translated into prognostic markers in some prospective studies; for instance, a 21-gene signature was recently developed for breast cancer (the Oncotype DX breast cancer assay) [37]. To this end, we constructed a prognostic model for pancreatic cancer by analyzing the transcriptomes of a large number of pancreatic cancer patients across multiple datasets, and subsequently developed a four-gene risk score.

Since gene expression platforms are based on different analytical and data processing methods, it is often challenging to compare and integrate results from multiple datasets. In some previous studies, researchers have obtained integrated results by intersecting the results from different cohorts, which may lead to bias. Therefore, we used the robust rank aggregation method to screen for significantly altered genes across seven microarray datasets in an unbiased manner, and subsequently identified 542 DEGs that overlapped between the microarray datasets and the RNA-Seq datasets from TCGA and GTEx. A similar approach has been used to identify DEGs in prostate cancer [38] and bladder cancer patients [39], as well as in Pan-cancer [40] and multi-omics analyses [41]. The pancreatic-cancer-related DEGs were functionally enriched in the tumor immune microenvironment, with particular influence on the desmoplastic stroma, immune cell infiltration and perineural invasion, which contribute to cancer progression [5], metastasis [42] and chemotherapy resistance [43].

High-throughput data tend to be interpreted from a clinical transformation perspective in the precision oncology era [44, 45]. It is necessary to integrate all the available information to identify the most relevant markers in a critical and comprehensive analysis. WGCNA is a powerful bioinformatics tool that detects clusters of functionally correlated genes and therefore can identify clinically relevant markers [13, 46]. WGCNA has been successfully used to identify molecular signatures in brain cells with distinct spatial distributions [47], to determine the key factors promoting hepatic ischemia-reperfusion injury [46] and to demarcate the molecular subtypes of pancreatic cancer [13]. The LASSO regression algorithm is another genotype-to-phenotype “bridge” that has been used to construct prognostic models from key radiomic [48] and immunohistochemical [49] features. Using these approaches, we found that overall survival was the main clinical trait associated with the transcriptome profiles of pancreatic cancer patients, and that *DSG3*, *ARNTL2*, *NUSAP1* and *KRT7* were independent prognostic factors.

Desmoglein 3 (DSG3) is a component of desmosomes, the button-like structures in the cytomembrane that facilitate intercellular and cell-to-matrix adhesion. DSG3 is overexpressed in head and neck cancer, where it functions as an oncogene [50, 51]. Previous studies have demonstrated that DSG3 is an accurate biomarker for staging sentinel lymph nodes in head and neck cancer [52, 53], and for distinguishing lung squamous cell carcinoma from other subtypes of lung cancer [54]. Similarly, high expression of the pro-metastatic transcription factor ARNTL2 predicts poor survival in lung adenocarcinoma [55]. Forced expression of *Arntl2* in estrogen receptor-negative breast cancer cells was found to increase their metastatic potential and thus portend a poor prognosis [56]. NUSAP1 promotes mitosis, cell cycle progression and the DNA damage response as a substrate of Cyclin F [57–59]. Overexpression of NUSAP1 correlates with poor survival in melanoma [60], cervical carcinoma [61], prostate cancer [62] and glioblastoma multiforme [63]. KRT7, a membrane-cytoskeleton linker required for cell adhesion, is overexpressed in colon cancer [64] and esophageal squamous cell carcinoma [65], and is associated with poor survival and metastasis in colon cancer [64]. In an *in vivo* model, KRT7 was found to promote the transition of basal cells into the multi-layered epithelium and Barrett’s esophagus [66]. Thus, all of the above genes have displayed pro-metastatic effects associated with poor outcomes in multiple cancers.

According to the transparent reporting of a multivariable prediction model for individual prognosis or diagnosis (TRIPOD) statement, internal validation (also called self-validation) is a necessary part of model development, and external validation to evaluate the performance of a model with other datasets is strongly recommended [67]. Therefore, we formulated our four-gene risk score based on the expression and Cox regression coefficient of each gene. Our model stratified pancreatic cancer patients of three independent cohorts into high- and low-risk groups with moderate accuracy. This three-cohort validation, together with the fact that our study was conducted with nine pancreatic cancer datasets in a platform-independent manner, indicates that our model is compatible across different platforms.

Several bioinformatic investigations in pancreatic cancer have previously been conducted from different perspectives. A nine-gene signature (*MET*, *KLK10*, *COL17A1*, *CEP55*, *ANKRD22*, *ITGB6*, *ARNTL2*, *MCOLN3* and *SLC25A45*) [68] and a four-gene signature (*LYRM1*, *KNTC1*, *IGF2BP2* and *CDC6*) [69] were proposed to predict the overall survival of pancreatic cancer patients. Four of the genes identified by these two studies (*MET*, *CEP55*, *ITGB6* and *ARNTL2*) were also identified as prognosis-associated genes in our study. The AUCs for three-year overall survival prediction with the previously reported nine-gene model were 0.621, 0.814 and 0.670 in one training and two external validation cohorts, respectively, while the AUCs for three-year overall survival prediction with our four-gene model were 0.839, 0.695, 0.747 and 0.872 (the latter two are data not shown) in one training, one internal and two external validation cohorts, respectively. Thus, our four-gene prognostic model achieved moderate accuracy with less complexity than the nine-gene model.

On the other hand, certain proposed models, such as a five-gene [70] or 25-gene signature [71], were developed through the comparison of gene expression profiles between long-term and short-term survival groups of pancreatic cancer patients. In these studies, genes involved in extracellular matrix organization, cell adhesion and immune response suppression tended to be activated in the short-term survival group, consistent with our findings (Figures 4 and 7B). Other previous bioinformatics studies included only one or two PDAC datasets, and thus could not characterize common prognosis-associated genes across various datasets [72–74]. In contrast, our risk score was based on multiple datasets, a significantly larger number of patients and both microarray and sequencing platforms. This difference in patient groups and data processing methods may account for the different results.

This was the first study to combine comprehensive pancreatic cancer cohorts in a systematic analysis strategy. The major strengths of this study were the performance of quality control measures, the cross-validation of the results and the use of multiple cohorts for consistency. The use of publicly available data from millions of assays is a challenge [75], and one prerequisite is retaining the quality of the raw data. Since issues with technique or RNA degradation may occur, we checked the quality of the enrolled cases and removed any cases with potential problems. Another concern when integrating different platforms is balancing the different batch effects or detection methods, so we adopted robust rank aggregation to perform an unbiased analysis.

Our prognostic model, which was derived from multiple cohorts and validated by three cohorts, deserves further validation and translation into clinical practice, since the four genes in our model encode proteins and can be examined in clinical practice through routine cost-effective methods. However, since this model was developed from microarray and sequencing expression profiles, more common and practical methods (e.g., quantitative real-time PCR or immunohistochemistry) need to be used to translate the model into clinical practice. Additional studies are needed to elucidate whether the protein levels of these genes are consistent with their transcriptional levels in pancreatic cancer. For oncological research, these consistently altered genes are worthy of further investigation, and the prognosis-associated genes should be further characterized for both their involvement in cancer progression and their value as therapeutic targets.

In conclusion, we constructed a four-gene prognostic model for pancreatic cancer that can predict post-surgical prognosis with moderate accuracy and facilitate therapeutic decision-making and clinical monitoring.

## MATERIALS AND METHODS

### Study design and cohorts

In order to avoid biases caused by single or small numbers of cohorts, such as those based on a specific race, detection method or analysis technique, here we conducted a systematic retrospective analysis. We screened all the available high-throughput Affymetrix microarray datasets (up to August 2, 2019) in the GEO database to identify datasets that included both normal and cancerous pancreatic tissues and passed our quality control assessment for all the enrolled raw data. The analysis strategy is summarized in Supplementary Figure 1. Ultimately, we enrolled two RNA-Seq cohorts and seven microarray datasets. We first sought to identify common DEGs across all nine enrolled cohorts. WGCNA was then performed to connect clinical traits with pancreatic cancer expression profiles in TCGA. Based on the results of the WGCNA, we focused on determining the significance of the DEGs in predicting post-operation overall survival.

To establish the molecular prognostic model, we combined the prognosis-associated genes from TCGA and GSE62452 in a univariate Cox regression analysis. Next, the intersecting results were shrunk with the LASSO regression algorithm, such that highly interconnected genes were alternated to avoid overfitting. The filtered genes were entered into the multivariate Cox regression analysis, and a scoring model was built to predict overall survival.

The cohort from TCGA was randomly divided into two comparable sub-cohorts (each N = 88). The training cohort was used to optimize the parameters in the LASSO and multivariate Cox regression analyses to build the risk score model, while the validation cohort was used to self-validate its performance. Two independent GEO microarray cohorts (GSE28735 and GSE62452) were also used to further validate the prognostic prediction model.

### Data acquisition

RNA-Seq data and clinical information from pancreatic cancer patients were downloaded from GTEx [26, 27] and TCGA [25, 76] via the UCSC Xena platform (https://xena.ucsc.edu/) [28]. The normal pancreatic and pancreatic-cancer-related RNA-Seq datasets are named ‘GTEX_RSEM_gene_fpkm’ and ‘TCGA-PAAD/ Xena_Metrices/TCGA-PAAD.htseq_fpkm’, while the downloaded clinical data are entitled ‘GTEX_ phenotype’ and ‘TCGA-PAAD/Xena_Metrices/TCGA-PAAD.GDC_phenotype’. In addition, seven pancreatic cancer microarray datasets – GSE32676 [77], GSE16515 [78], GSE71989 [79], GSE41368 [80], GSE15471 [81], GSE28735 [82, 83] and GSE62452 [84] – were downloaded from the GEO database [85]. The human TF list was retrieved from the Cistrome Cancer website (http://cistrome.org/CistromeCancer/CancerTarget/) [31], and the list of human protein kinases was obtained from the Kinome of *Homo sapiens* [32].

### Microarray raw data quality control and identification of DEGs

Since all the selected GEO datasets were based on the Affymetrix platform, quality control was performed with Transcriptome Analysis Console software (Applied Biosystems, version 4.0.2) and the R ‘simpleAffy’ [23] and ‘Affy’ [22] packages. After averaging the expression values of the genes corresponding to the multi-microarray probes, we calculated the log_2_^FC^ ratios between the normal and tumorous samples in each dataset, and determined their statistical significance with the R ‘limma’ package [86]. The ‘RobustRankAggreg’ R package was then used to identify the DEGs across the multi-microarray datasets based on a prioritized gene list, with a numerical core and *p* value determined through Bonferroni correction [24].

### TCGA and GTEx sequencing data integration and DEG identification

After the UCSC Toil RNA-Seq Recompute data were downloaded, the FPKM (fragments per kilobase of transcript per million mapped reads) values from GTEx were log_2_^(x+0.001)^ transformed, and the values from TCGA were log_2_^(x+1)^ transformed. Both forms were unified as log_2_^(x+1)^, and the ‘limma’ package was used to screen for DEGs between normal and tumorous pancreatic tissues, with a |log_2_^FC^| > 1 and a false discovery rate < 0.05 as the cut-offs. For both the sequencing and microarray platforms, a log_2_^FC^ > 0 indicated gene overexpression in the tumor tissues.

### Construction of the heatmap and GO plot

The DEGs in each sample were plotted with the R ‘pheatmap’ package [87]. Entrez gene annotations were referred to ‘org.Hs.eg.db’ (Carlson M, 2019. org.Hs.eg.db: Genome wide annotation for Human. R package version 3.8.2.), and the GO analysis was performed in the R ‘clusterProfiler’ package [88] with an adjusted *p* value < 0.01 and a *q* value < 0.01 as the cut-offs. The GO cluster was plotted with the R ‘GOplot’ package [89].

### WGCNA

The R ‘WGCNA’ package was used to detect gene modules and evaluate the correlation of each module with clinico-pathological factors. To be specific, (i) we extracted data from TCGA on pancreatic cancer patients with complete clinical data regarding age, gender, tumor histological grade, clinical stage, TNM classification (except for M, as there were numerous cases lacking metastasis information), overall survival rate and duration, along with their gene expression profiles; (ii) sample clustering was performed to detect any outliers; (iii) the power (soft thresholding) β value was set to 10 so that we could achieve a scale-free topology fit index (scale-free R^2^) greater than 0.9 and maintain optimal mean connectivity; (iv) the adjacency matrix was transformed into a topological overlap matrix (TOM) to define gene co-expression similarity; (v) the ‘hclust’ algorithm was used to create a gene hierarchical clustering based on the TOM dissimilarity measure; (vi) the optimal module size was set to 30, and a dynamic tree cut was used to identify the modules; (vii) after the dissimilarity of the module eigengenes was calculated, the similarity cut-off was set to 0.75 in order to merge the modules; (viii) since we had already identified the featured gene expression profiles for each module and the clinical traits of the patients, the correlations of the module eigengenes with the clinico-pathological factors were determined.

### Construction of the prognostic model

Prognosis-associated genes were identified by the R ‘survival’ package [90] as those impacting overall survival with HRs and 95% CIs greater than or less than 1. Identified genes were then subjected to univariate Cox regression analysis with *p* < 0.05 as the significance threshold. The list was further narrowed down by the LASSO algorithm with the R ‘glmnet’ package [91], and the optimal tuning parameter (λ) was chosen to achieve the minimal partial likelihood deviance in the cross-validation plot. The genes still harboring non-zero corresponding coefficients were entered into the multivariate Cox model. Finally, the expression of each gene was multiplied by its Cox regression coefficient, and these values were summed to calculate the risk score.

### Training and validation of the prognostic model

The R ‘caret’ package [92] was used to divide the cohort from TCGA randomly into training and self-validation sets (N = 88 each). Two microarray datasets (GSE28735 and GSE62452) were selected as independent validation cohorts. The risk score was calculated for each patient, and the patients were then divided into high-risk and low-risk groups based on the median risk score of the training cohort. The performance of the model was evaluated in terms of its ability to predict one-year and three-year overall survival in the high- and low-risk groups.

### Statistical analysis

Continuous variables with normal distributions are reported as the mean + standard deviation, while those with skewed distributions are reported as the median (25^th^ percentile - 75^th^ percentile). Categorical variables are reported as frequencies (proportions). All analyses were conducted with the R foundation for statistical computing (version 3.6.1). Pearson correlation analysis was used to determine the correlation between a module eigengene and a clinico-pathological factor, with *p* < 0.05 indicating statistical significance. Kaplan-Meier survival analysis and the log-rank test were performed with the R ‘survival’ package. The time-dependent ROC curve from censored survival data was plotted with the R ‘survivalROC’ package [93].

## Supplementary Material

Supplementary Figures

Supplementary Table 1

Supplementary Table 2
