# Supplementary material for: Development of a four-gene prognostic model for pancreatic cancer based on transcriptome dysregulation
Source: Aging (Albany NY). 2020 Feb 20;12(4):3747–70. doi: 10.18632/aging.102844 (PMC7066910; doi:10.18632/aging.102844)
Supplement: Supplementary Figures [file aging-12-102844-s002..pdf]

SUPPLEMENTARY FIGURES

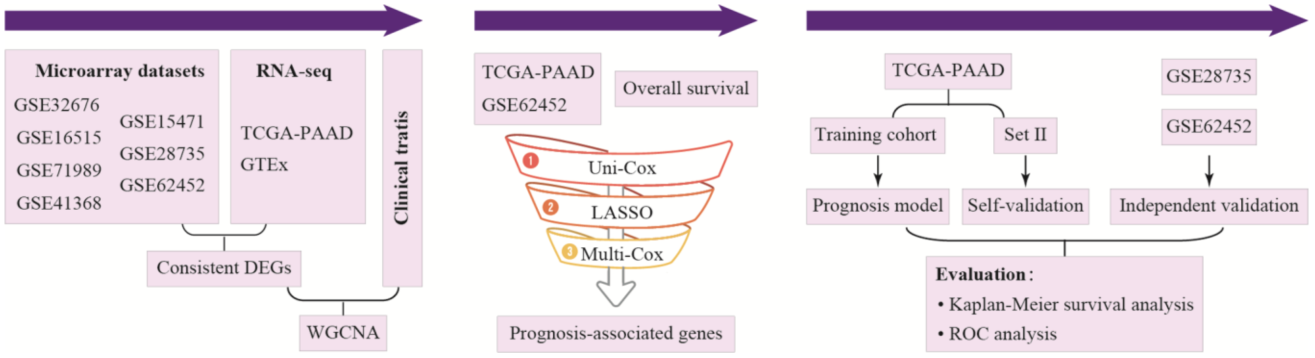

Supplementary Figure 1. Flowchart presenting the study design of this retrospective analysis. "Uni-Cox" refers to univariate Cox regression, and "Multi-Cox" refers to multivariate Cox model construction.

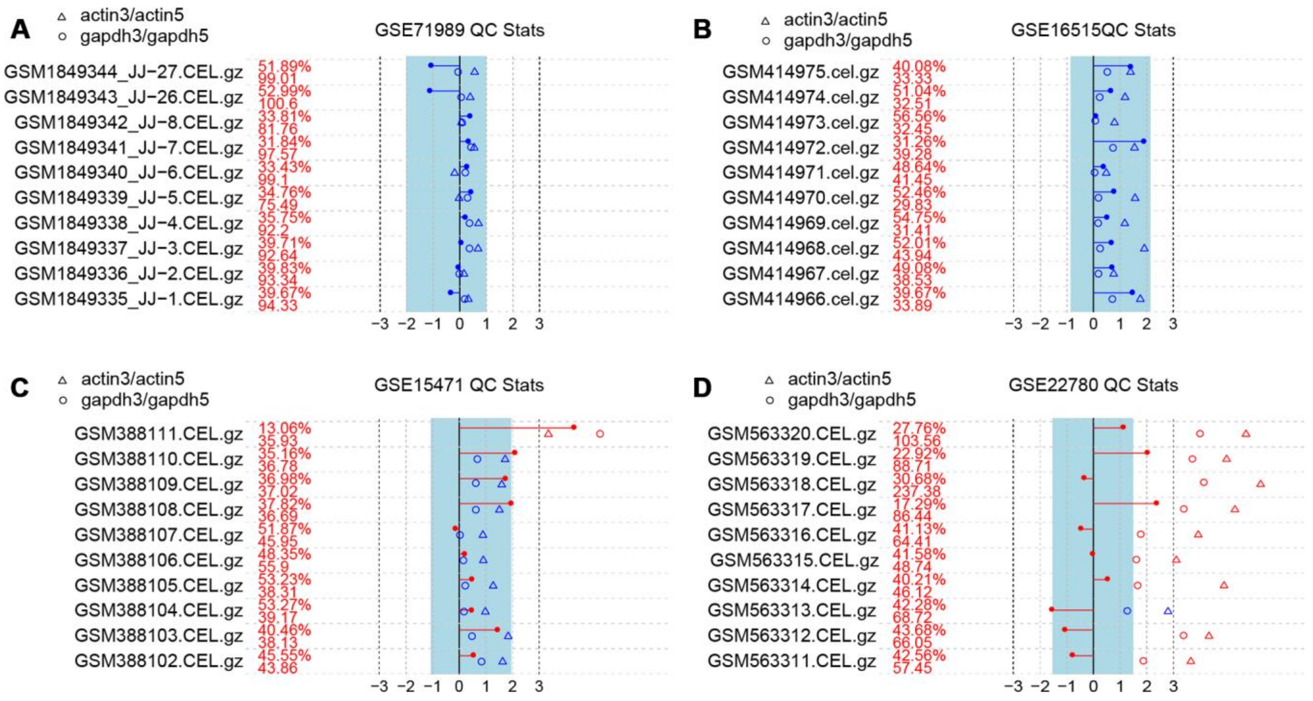

Supplementary Figure 2. Quality control results from four pancreatic cancer microarrays. For each panel, the raw data names of each case are presented in the first column, the % present calls (top) and average background (bottom) are displayed in the second column, and the range within which the scale factor should be located (a horizontal line with a dot at the top) is indicated by the blue stripe. The 3'-to-5' ratios of *β-actin* and *GAPDH* are plotted as triangles and circles, respectively. Outliers of the ratio are shown in red, and otherwise are shown in blue. The representative results of 10 samples in each array demonstrate the all-passed (**A** and **B**), one outlier case (**C**) and all-failed (**D**) samples in our quality control recheck.

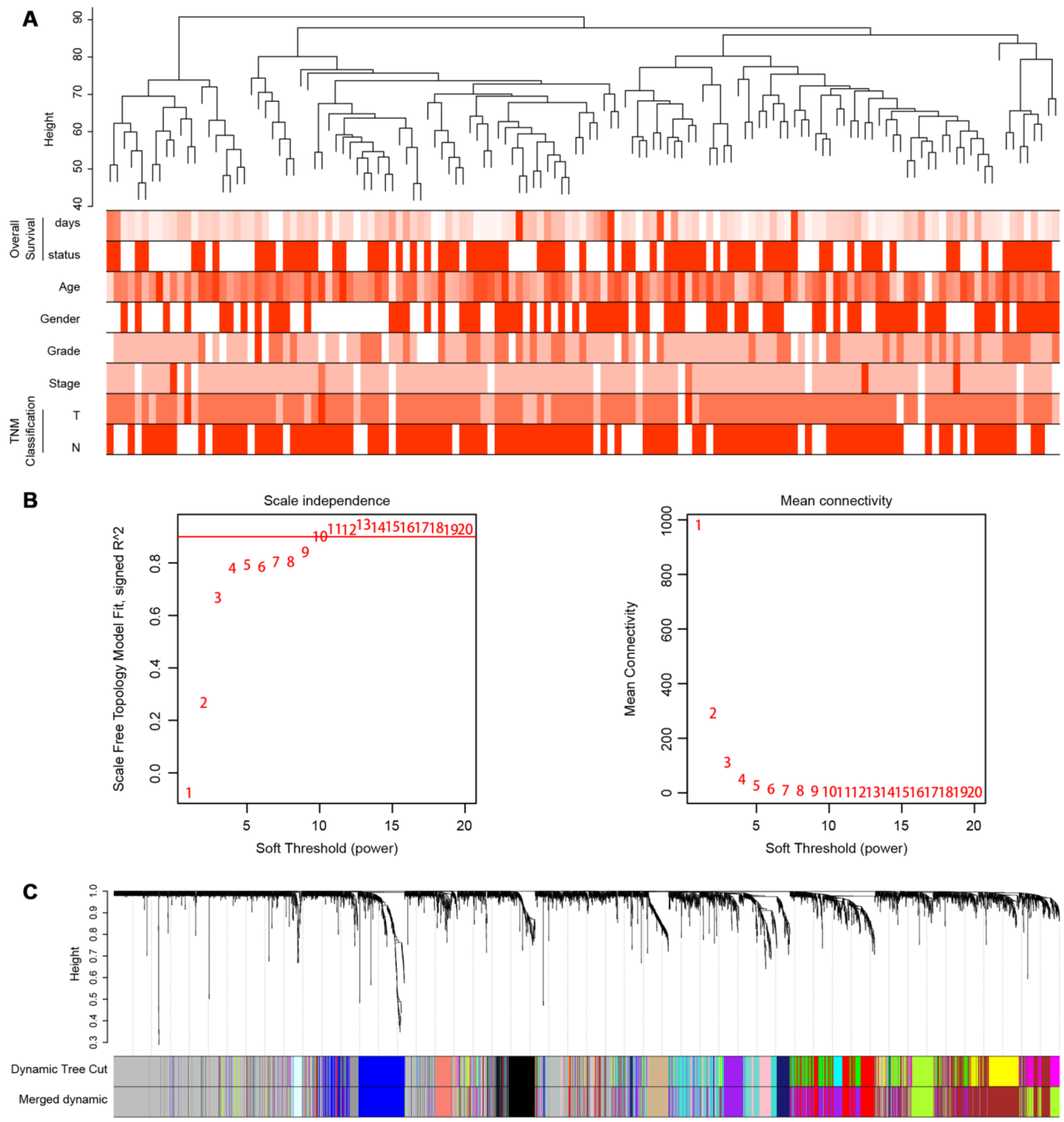

Supplementary Figure 3. Modules of TCGA pancreatic cancer expression profiles (N = 135). (**A**) Sample dendrogram and clinical trait heatmap. With the exception of four outliers, all the samples could be hierarchically clustered. Binary variables (overall survival status, gender) are presented as red or white blocks, and continuous variables (overall survival days, age, grade, stage, T, N) are presented as color-coded blocks, with the color saturation corresponding to the value of the variable. (**B**) Determination of the soft-thresholding power for the optimal scale-free topology fit index (scale-free R^2) (left) and mean connectivity (right). The red horizontal line represents $R^2 = 0.9$. (**C**) Module identification. The dendrogram represents the gene clustering based on the TOM dissimilarity measure. Genes with relative interrelatedness are located on the same or neighboring branches. A dynamic tree cut at module size 30 resulted in 17 color-coded modules. By merging the modules after calculating the dissimilarity of the module eigengenes at a cut-off of 0.75, we identified 14 modules.
