# Supplementary material for: Development of a four-gene prognostic model for pancreatic cancer based on transcriptome dysregulation
Source: Aging (Albany NY). 2020 Feb 20;12(4):3747–70. doi: 10.18632/aging.102844 (PMC7066910; doi:10.18632/aging.102844)
Supplement: Supplementary Table 1 [file aging-12-102844-s001..docx]

**Supplementary Table 1.** **The 542 differentially expressed genes in both the sequencing and multi-microarray datasets.**

| Gene | Seven GEO datasets | | | TCGA combined GTEx datasets | | |
| --- | --- | --- | --- | --- | --- | --- |
|  | Log_2_^FC^ | *p* value | Adjust *p* value | Log_2_^FC^ | *p* value | FDR |
| *CEACAM6* | 3.81 | 7.15E-23 | 1.79E-18 | 7.06 | 2.09E-50 | 8.70E-50 |
| *CTSE* | 3.62 | 1.87E-19 | 4.67E-15 | 6.71 | 9.55E-48 | 3.07E-47 |
| *TFF1* | 2.96 | 1.13E-14 | 2.83E-10 | 7.28 | 2.72E-49 | 9.94E-49 |
| *S100P* | 3.78 | 1.36E-14 | 3.40E-10 | 6.35 | 5.37E-48 | 1.76E-47 |
| *CEACAM5* | 2.94 | 6.24E-12 | 1.56E-07 | 6.18 | 9.91E-49 | 3.45E-48 |
| *MSLN* | 2.61 | 1.82E-10 | 4.56E-06 | 5.84 | 9.66E-50 | 3.70E-49 |
| *COL3A1* | 2.18 | 1.97E-11 | 4.92E-07 | 6.24 | 1.66E-52 | 9.95E-52 |
| *LAMC2* | 3.02 | 6.39E-14 | 1.60E-09 | 5.23 | 9.67E-52 | 4.91E-51 |
| *KRT19* | 2.38 | 1.51E-12 | 3.77E-08 | 5.81 | 2.64E-52 | 1.50E-51 |
| *S100A6* | 1.89 | 9.65E-13 | 2.41E-08 | 6.20 | 2.20E-57 | 1.73E-55 |
| *LAMB3* | 2.55 | 1.43E-11 | 3.57E-07 | 5.42 | 3.02E-50 | 1.23E-49 |
| *POSTN* | 3.07 | 1.50E-14 | 3.76E-10 | 4.90 | 2.18E-52 | 1.26E-51 |
| *TSPAN1* | 2.91 | 1.62E-14 | 4.05E-10 | 4.97 | 4.50E-55 | 8.90E-54 |
| *CST1* | 2.92 | 2.68E-15 | 6.70E-11 | 4.88 | 7.01E-56 | 2.10E-54 |
| *MMP1* | 2.66 | 7.83E-15 | 1.96E-10 | 5.14 | 8.29E-53 | 5.40E-52 |
| *SFN* | 2.11 | 1.18E-10 | 2.94E-06 | 5.67 | 1.13E-49 | 4.28E-49 |
| *COL1A1* | 1.61 | 4.80E-09 | 0.000 | 6.15 | 3.06E-52 | 1.71E-51 |
| *C19orf33* | 2.19 | 1.25E-08 | 0.000 | 5.51 | 1.04E-49 | 3.97E-49 |
| *LCN2* | 2.68 | 1.02E-14 | 2.55E-10 | 5.00 | 3.51E-44 | 9.13E-44 |
| *COL10A1* | 2.79 | 6.14E-15 | 1.54E-10 | 4.67 | 1.79E-50 | 7.48E-50 |
| *CLDN18* | 2.72 | 1.29E-13 | 3.22E-09 | 4.71 | 1.33E-37 | 2.73E-37 |
| *TCN1* | 2.55 | 1.43E-13 | 3.57E-09 | 4.86 | 2.45E-45 | 6.74E-45 |
| *SLPI* | 2.34 | 2.97E-12 | 7.44E-08 | 5.01 | 1.27E-51 | 6.33E-51 |
| *TFF2* | 1.92 | 2.81E-08 | 0.001 | 5.37 | 1.28E-43 | 3.26E-43 |
| *AGR2* | 2.39 | 1.61E-11 | 4.02E-07 | 4.88 | 9.31E-50 | 3.57E-49 |
| *COL1A2* | 2.39 | 4.39E-12 | 1.10E-07 | 4.75 | 3.80E-50 | 1.53E-49 |
| *THBS2* | 2.41 | 5.15E-12 | 1.29E-07 | 4.65 | 3.14E-52 | 1.75E-51 |
| *CTHRC1* | 2.40 | 1.44E-10 | 3.60E-06 | 4.60 | 5.38E-53 | 3.73E-52 |
| *SULF1* | 3.07 | 8.63E-14 | 2.16E-09 | 3.93 | 3.23E-51 | 1.51E-50 |
| *SFRP2* | 1.68 | 4.50E-07 | 0.011 | 5.32 | 5.49E-47 | 1.67E-46 |
| *SLC6A14* | 3.86 | 6.95E-21 | 1.74E-16 | 3.03 | 3.61E-49 | 1.31E-48 |
| *MMP11* | 1.58 | 1.22E-08 | 0.000 | 5.27 | 5.56E-53 | 3.85E-52 |
| *LYZ* | 1.54 | 8.25E-09 | 0.000 | 5.27 | 3.85E-49 | 1.39E-48 |
| *GPRC5A* | 2.19 | 5.38E-14 | 1.35E-09 | 4.61 | 3.49E-48 | 1.16E-47 |
| *TMPRSS4* | 2.80 | 1.29E-11 | 3.24E-07 | 3.99 | 9.48E-50 | 3.63E-49 |
| *GABRP* | 2.92 | 3.42E-16 | 8.56E-12 | 3.75 | 4.61E-47 | 1.41E-46 |
| *GJB2* | 2.62 | 6.36E-13 | 1.59E-08 | 4.00 | 1.71E-46 | 5.05E-46 |
| *COL5A2* | 2.26 | 8.78E-12 | 2.20E-07 | 4.35 | 6.28E-55 | 1.17E-53 |
| *CXCL5* | 2.42 | 6.07E-16 | 1.52E-11 | 4.14 | 3.56E-46 | 1.03E-45 |
| *GPX2* | 1.96 | 8.57E-10 | 2.14E-05 | 4.53 | 1.79E-50 | 7.48E-50 |
| *BGN* | 1.59 | 1.65E-08 | 0.000 | 4.85 | 9.52E-54 | 9.18E-53 |
| *IGFBP3* | 1.91 | 1.41E-11 | 3.53E-07 | 4.28 | 4.08E-54 | 4.82E-53 |
| *PSCA* | 1.49 | 7.53E-07 | 0.019 | 4.66 | 2.56E-39 | 5.52E-39 |
| *COMP* | 1.92 | 1.13E-09 | 2.82E-05 | 4.21 | 1.22E-45 | 3.43E-45 |
| *NQO1* | 2.32 | 8.13E-15 | 2.04E-10 | 3.79 | 5.04E-53 | 3.52E-52 |
| *IFI6* | 1.47 | 8.40E-11 | 2.10E-06 | 4.64 | 3.06E-54 | 3.94E-53 |
| *TMSB10* | 1.29 | 2.47E-09 | 6.18E-05 | 4.75 | 1.44E-56 | 6.77E-55 |
| *APOL1* | 1.46 | 2.34E-09 | 5.84E-05 | 4.57 | 2.44E-56 | 9.70E-55 |
| *PI3* | 1.75 | 1.97E-10 | 4.92E-06 | 4.26 | 3.07E-48 | 1.03E-47 |
| *GCNT3* | 2.34 | 3.12E-14 | 7.82E-10 | 3.61 | 1.07E-49 | 4.09E-49 |
| *S100A11* | 1.52 | 1.23E-09 | 3.08E-05 | 4.43 | 2.24E-51 | 1.07E-50 |
| *COL11A1* | 2.70 | 3.01E-14 | 7.54E-10 | 3.23 | 2.25E-43 | 5.65E-43 |
| *MUC13* | 1.67 | 6.03E-09 | 0.000 | 4.23 | 3.33E-52 | 1.84E-51 |
| *FN1* | 2.53 | 7.58E-13 | 1.90E-08 | 3.35 | 1.74E-41 | 4.05E-41 |
| *INHBA* | 2.52 | 3.65E-14 | 9.13E-10 | 3.33 | 1.42E-54 | 2.18E-53 |
| *TIMP1* | 1.69 | 3.04E-08 | 0.001 | 4.15 | 9.29E-54 | 9.00E-53 |
| *SFRP4* | 1.85 | 5.05E-08 | 0.001 | 3.98 | 4.50E-49 | 1.61E-48 |
| *VSIG2* | 1.23 | 1.43E-06 | 0.036 | 4.57 | 3.34E-51 | 1.55E-50 |
| *VCAN* | 2.54 | 6.79E-13 | 1.70E-08 | 3.24 | 7.32E-49 | 2.58E-48 |
| *MMP7* | 2.08 | 4.00E-12 | 1.00E-07 | 3.70 | 1.60E-39 | 3.47E-39 |
| *COL5A1* | 1.81 | 3.92E-09 | 9.81E-05 | 3.96 | 6.04E-51 | 2.69E-50 |
| *ITGA2* | 2.39 | 1.00E-12 | 2.51E-08 | 3.38 | 3.25E-56 | 1.22E-54 |
| *SPARC* | 1.63 | 1.54E-08 | 0.000 | 4.11 | 2.02E-52 | 1.18E-51 |
| *AOC1* | 1.81 | 7.13E-07 | 0.018 | 3.86 | 4.01E-54 | 4.76E-53 |
| *CTSK* | 1.73 | 1.51E-08 | 0.000 | 3.91 | 3.37E-53 | 2.51E-52 |
| *PLAU* | 1.66 | 1.64E-10 | 4.11E-06 | 3.96 | 3.94E-53 | 2.86E-52 |
| *DUOX2* | 2.14 | 9.68E-12 | 2.42E-07 | 3.48 | 7.03E-43 | 1.73E-42 |
| *S100A14* | 1.57 | 4.23E-09 | 0.000 | 4.04 | 3.07E-46 | 8.94E-46 |
| *SLC44A4* | 1.69 | 8.23E-11 | 2.06E-06 | 3.86 | 2.60E-50 | 1.06E-49 |
| *SDR16C5* | 2.61 | 9.24E-14 | 2.31E-09 | 2.93 | 1.01E-47 | 3.26E-47 |
| *TMC5* | 2.35 | 1.06E-14 | 2.64E-10 | 3.19 | 9.86E-48 | 3.16E-47 |
| *SERPINB5* | 2.17 | 3.79E-11 | 9.49E-07 | 3.35 | 5.74E-48 | 1.88E-47 |
| *SDC1* | 1.40 | 2.62E-09 | 6.55E-05 | 4.00 | 1.46E-53 | 1.28E-52 |
| *KCNN4* | 1.41 | 4.91E-09 | 0.000 | 3.95 | 1.36E-50 | 5.79E-50 |
| *OLFML2B* | 1.79 | 1.96E-09 | 4.90E-05 | 3.56 | 3.52E-54 | 4.37E-53 |
| *IGFBP5* | 1.32 | 5.84E-08 | 0.001 | 4.00 | 1.19E-50 | 5.10E-50 |
| *EPS8L3* | 1.60 | 2.11E-07 | 0.005 | 3.63 | 1.02E-47 | 3.26E-47 |
| *C15orf48* | 2.03 | 4.06E-11 | 1.02E-06 | 3.18 | 3.73E-49 | 1.35E-48 |
| *AEBP1* | 1.60 | 1.57E-09 | 3.92E-05 | 3.61 | 4.23E-49 | 1.52E-48 |
| *PLAT* | 1.74 | 3.66E-11 | 9.17E-07 | 3.45 | 3.91E-52 | 2.13E-51 |
| *KLK6* | 1.35 | 3.47E-07 | 0.009 | 3.85 | 4.48E-49 | 1.61E-48 |
| *LUM* | 1.36 | 1.22E-06 | 0.031 | 3.82 | 3.49E-47 | 1.08E-46 |
| *S100A16* | 1.36 | 1.49E-10 | 3.73E-06 | 3.81 | 5.29E-53 | 3.68E-52 |
| *ASPN* | 1.52 | 6.03E-08 | 0.002 | 3.59 | 2.44E-52 | 1.39E-51 |
| *MUCL3* | 1.02 | 1.07E-06 | 0.027 | 4.07 | 1.08E-38 | 2.29E-38 |
| *CCL18* | 1.59 | 7.33E-11 | 1.83E-06 | 3.48 | 9.15E-54 | 8.92E-53 |
| *CCL20* | 2.34 | 5.73E-15 | 1.43E-10 | 2.72 | 1.48E-43 | 3.74E-43 |
| *ACTB* | 1.05 | 6.20E-08 | 0.002 | 3.97 | 1.12E-55 | 3.04E-54 |
| *FOXQ1* | 1.61 | 5.62E-09 | 0.000 | 3.39 | 1.54E-51 | 7.57E-51 |
| *LAMP5* | 1.64 | 1.15E-10 | 2.89E-06 | 3.36 | 1.52E-52 | 9.20E-52 |
| *MMP12* | 2.50 | 6.20E-15 | 1.55E-10 | 2.46 | 5.01E-57 | 3.09E-55 |
| *SLC2A1* | 1.48 | 4.64E-07 | 0.012 | 3.48 | 3.22E-55 | 6.88E-54 |
| *ACSL5* | 1.67 | 1.09E-10 | 2.72E-06 | 3.29 | 9.31E-50 | 3.57E-49 |
| *CDH3* | 1.88 | 1.96E-09 | 4.90E-05 | 3.06 | 1.07E-49 | 4.09E-49 |
| *COL12A1* | 2.00 | 1.23E-11 | 3.08E-07 | 2.93 | 1.84E-48 | 6.28E-48 |
| *FNDC1* | 2.02 | 2.14E-12 | 5.37E-08 | 2.90 | 1.03E-48 | 3.59E-48 |
| *CAMK2N1* | 1.19 | 5.98E-10 | 1.50E-05 | 3.71 | 5.53E-58 | 5.47E-56 |
| *SFTA2* | 1.92 | 1.55E-08 | 0.000 | 2.97 | 9.12E-40 | 2.00E-39 |
| *TSPAN8* | 1.70 | 2.43E-10 | 6.08E-06 | 3.17 | 1.01E-43 | 2.57E-43 |
| *S100A2* | 1.92 | 9.75E-10 | 2.44E-05 | 2.94 | 1.44E-44 | 3.80E-44 |
| *VSIG1* | 2.02 | 4.74E-10 | 1.19E-05 | 2.83 | 4.62E-45 | 1.25E-44 |
| *AGR3* | 1.86 | 1.48E-10 | 3.70E-06 | 2.99 | 6.30E-43 | 1.55E-42 |
| *ANTXR1* | 1.41 | 2.04E-07 | 0.005 | 3.44 | 4.57E-53 | 3.23E-52 |
| *ST6GALNAC1* | 1.88 | 5.75E-10 | 1.44E-05 | 2.96 | 3.31E-46 | 9.63E-46 |
| *BST2* | 1.11 | 1.47E-07 | 0.004 | 3.71 | 4.24E-53 | 3.05E-52 |
| *HLA-A* | 1.01 | 1.92E-07 | 0.005 | 3.81 | 1.63E-55 | 4.05E-54 |
| *PLAC8* | 2.32 | 4.38E-14 | 1.10E-09 | 2.47 | 2.93E-50 | 1.19E-49 |
| *SEMA3C* | 1.71 | 1.30E-09 | 3.24E-05 | 3.07 | 4.67E-51 | 2.13E-50 |
| *GALNT5* | 2.03 | 1.17E-08 | 0.000 | 2.74 | 4.63E-52 | 2.49E-51 |
| *DPYSL3* | 1.30 | 1.05E-09 | 2.63E-05 | 3.47 | 2.14E-54 | 3.00E-53 |
| *DKK1* | 2.29 | 1.65E-12 | 4.13E-08 | 2.47 | 1.36E-42 | 3.29E-42 |
| *MXRA5* | 1.73 | 9.83E-13 | 2.46E-08 | 3.03 | 6.46E-47 | 1.96E-46 |
| *MMP9* | 1.02 | 2.74E-07 | 0.007 | 3.73 | 1.91E-54 | 2.76E-53 |
| *RAB31* | 1.77 | 2.49E-09 | 6.24E-05 | 2.98 | 3.85E-52 | 2.10E-51 |
| *LGALS3BP* | 1.24 | 3.56E-10 | 8.92E-06 | 3.50 | 2.09E-57 | 1.67E-55 |
| *KLK7* | 1.41 | 3.35E-07 | 0.008 | 3.32 | 2.06E-41 | 4.77E-41 |
| *TMEM45B* | 1.60 | 3.57E-08 | 0.001 | 3.09 | 7.66E-55 | 1.36E-53 |
| *LGALS4* | 1.58 | 7.45E-09 | 0.000 | 3.11 | 2.51E-39 | 5.41E-39 |
| *COL8A1* | 1.60 | 4.17E-09 | 0.000 | 3.08 | 9.69E-50 | 3.71E-49 |
| *CAPN8* | 2.01 | 1.82E-07 | 0.005 | 2.66 | 9.78E-36 | 1.91E-35 |
| *CKS2* | 1.47 | 1.42E-09 | 3.55E-05 | 3.18 | 3.79E-57 | 2.62E-55 |
| *ANO1* | 1.56 | 2.77E-10 | 6.93E-06 | 3.09 | 5.87E-54 | 6.37E-53 |
| *KLF5* | 1.46 | 1.79E-07 | 0.004 | 3.17 | 7.69E-48 | 2.49E-47 |
| *S100A4* | 1.44 | 1.45E-07 | 0.004 | 3.19 | 1.72E-46 | 5.09E-46 |
| *AKR1B10* | 1.43 | 1.93E-09 | 4.82E-05 | 3.19 | 8.83E-42 | 2.08E-41 |
| *EFNB2* | 1.59 | 1.19E-12 | 2.97E-08 | 3.03 | 2.09E-57 | 1.67E-55 |
| *TNFRSF21* | 1.15 | 9.07E-09 | 0.000 | 3.41 | 1.29E-55 | 3.37E-54 |
| *OLR1* | 2.24 | 1.30E-12 | 3.26E-08 | 2.31 | 5.57E-40 | 1.23E-39 |
| *CST2* | 1.25 | 1.21E-08 | 0.000 | 3.29 | 2.19E-51 | 1.05E-50 |
| *MATN3* | 1.60 | 1.41E-11 | 3.53E-07 | 2.93 | 2.04E-52 | 1.19E-51 |
| *HK2* | 1.56 | 6.93E-10 | 1.73E-05 | 2.97 | 9.30E-51 | 4.03E-50 |
| *NMU* | 1.94 | 5.97E-11 | 1.49E-06 | 2.58 | 2.45E-53 | 1.93E-52 |
| *S100A10* | 1.21 | 9.77E-09 | 0.000 | 3.30 | 2.69E-52 | 1.52E-51 |
| *EDNRA* | 1.87 | 2.15E-10 | 5.38E-06 | 2.63 | 2.75E-53 | 2.11E-52 |
| *GREM1* | 2.03 | 3.88E-11 | 9.70E-07 | 2.46 | 8.07E-41 | 1.83E-40 |
| *HLA-B* | 1.10 | 3.34E-08 | 0.001 | 3.39 | 1.33E-53 | 1.19E-52 |
| *CORO2A* | 1.32 | 4.21E-08 | 0.001 | 3.16 | 5.82E-58 | 5.66E-56 |
| *ANXA10* | 2.40 | 1.77E-12 | 4.43E-08 | 2.08 | 8.93E-19 | 1.26E-18 |
| *ECM1* | 1.33 | 4.06E-10 | 1.02E-05 | 3.13 | 2.27E-52 | 1.31E-51 |
| *SULF2* | 1.73 | 2.56E-10 | 6.41E-06 | 2.71 | 1.16E-51 | 5.85E-51 |
| *IFI27* | 2.12 | 6.11E-13 | 1.53E-08 | 2.31 | 6.28E-40 | 1.38E-39 |
| *CD55* | 1.43 | 3.62E-10 | 9.06E-06 | 2.99 | 2.68E-54 | 3.55E-53 |
| *LGALS1* | 1.37 | 8.28E-08 | 0.002 | 3.03 | 2.75E-47 | 8.56E-47 |
| *MYOF* | 1.69 | 3.01E-11 | 7.53E-07 | 2.71 | 2.80E-51 | 1.32E-50 |
| *TRIM29* | 1.67 | 8.11E-08 | 0.002 | 2.71 | 1.73E-37 | 3.53E-37 |
| *TOP2A* | 1.84 | 1.78E-11 | 4.45E-07 | 2.52 | 1.60E-53 | 1.37E-52 |
| *AHNAK2* | 2.10 | 4.60E-12 | 1.15E-07 | 2.25 | 1.07E-45 | 3.00E-45 |
| *KRT23* | 2.09 | 2.46E-12 | 6.15E-08 | 2.25 | 2.04E-36 | 4.06E-36 |
| *SGPP2* | 1.46 | 4.48E-09 | 0.000 | 2.87 | 2.49E-53 | 1.96E-52 |
| *DMBT1* | 1.30 | 4.67E-08 | 0.001 | 3.03 | 1.57E-34 | 2.97E-34 |
| *LIPH* | 1.51 | 3.17E-07 | 0.008 | 2.78 | 4.57E-50 | 1.82E-49 |
| *CAPG* | 1.75 | 6.22E-12 | 1.56E-07 | 2.53 | 2.17E-45 | 5.99E-45 |
| *EDIL3* | 1.44 | 2.69E-08 | 0.001 | 2.83 | 2.17E-56 | 8.98E-55 |
| *LY6E* | 1.39 | 6.82E-10 | 1.71E-05 | 2.86 | 1.59E-46 | 4.72E-46 |
| *TNFAIP6* | 1.93 | 2.99E-10 | 7.48E-06 | 2.32 | 2.69E-51 | 1.27E-50 |
| *CDH11* | 1.99 | 8.79E-11 | 2.20E-06 | 2.24 | 1.51E-45 | 4.21E-45 |
| *PLEK2* | 1.36 | 3.97E-07 | 0.010 | 2.86 | 3.36E-46 | 9.77E-46 |
| *ARL4C* | 1.04 | 7.84E-09 | 0.000 | 3.12 | 2.22E-51 | 1.06E-50 |
| *TPX2* | 1.37 | 8.01E-10 | 2.01E-05 | 2.79 | 6.53E-57 | 3.84E-55 |
| *TRIM31* | 1.47 | 1.50E-07 | 0.004 | 2.69 | 8.83E-45 | 2.36E-44 |
| *OAS1* | 1.79 | 3.25E-12 | 8.14E-08 | 2.36 | 1.97E-50 | 8.19E-50 |
| *AHR* | 1.16 | 7.41E-08 | 0.002 | 2.96 | 3.53E-56 | 1.30E-54 |
| *CXCL8* | 1.85 | 1.43E-08 | 0.000 | 2.26 | 3.34E-32 | 6.00E-32 |
| *OLFM4* | 1.33 | 1.13E-07 | 0.003 | 2.77 | 1.92E-16 | 2.62E-16 |
| *LAPTM5* | 1.04 | 1.72E-07 | 0.004 | 3.06 | 1.89E-49 | 7.03E-49 |
| *PLAUR* | 1.23 | 5.19E-09 | 0.000 | 2.87 | 8.88E-50 | 3.42E-49 |
| *NT5E* | 1.35 | 1.05E-10 | 2.62E-06 | 2.74 | 7.92E-55 | 1.40E-53 |
| *DHRS9* | 1.58 | 1.87E-09 | 4.67E-05 | 2.51 | 1.88E-45 | 5.22E-45 |
| *UBE2T* | 1.48 | 1.00E-09 | 2.51E-05 | 2.60 | 8.93E-58 | 8.48E-56 |
| *ANLN* | 2.15 | 2.43E-13 | 6.09E-09 | 1.92 | 3.45E-51 | 1.60E-50 |
| *VILL* | 1.50 | 4.77E-08 | 0.001 | 2.57 | 5.39E-48 | 1.77E-47 |
| *VNN1* | 1.30 | 7.22E-10 | 1.81E-05 | 2.76 | 2.23E-46 | 6.54E-46 |
| *AREG* | 1.05 | 5.05E-08 | 0.001 | 2.97 | 2.84E-50 | 1.16E-49 |
| *COL4A1* | 1.02 | 4.13E-08 | 0.001 | 2.99 | 2.48E-52 | 1.42E-51 |
| *MAL2* | 1.42 | 6.01E-08 | 0.002 | 2.59 | 6.03E-53 | 4.13E-52 |
| *LTBP1* | 1.16 | 1.68E-07 | 0.004 | 2.85 | 2.01E-52 | 1.17E-51 |
| *ZG16B* | 1.64 | 4.26E-07 | 0.011 | 2.35 | 1.23E-38 | 2.60E-38 |
| *SAMD9* | 1.56 | 4.53E-12 | 1.13E-07 | 2.42 | 3.82E-54 | 4.61E-53 |
| *BCAS1* | 1.51 | 3.65E-07 | 0.009 | 2.46 | 6.97E-47 | 2.11E-46 |
| *LIF* | 1.01 | 1.33E-06 | 0.033 | 2.94 | 2.36E-49 | 8.68E-49 |
| *CEMIP* | 1.45 | 1.51E-08 | 0.000 | 2.47 | 7.53E-52 | 3.88E-51 |
| *CLIC1* | 1.00 | 9.20E-10 | 2.30E-05 | 2.92 | 1.89E-56 | 8.18E-55 |
| *FERMT1* | 1.30 | 1.84E-06 | 0.046 | 2.62 | 4.82E-51 | 2.19E-50 |
| *CEACAM1* | 1.31 | 3.66E-07 | 0.009 | 2.61 | 2.68E-55 | 5.92E-54 |
| *CXCL10* | 1.62 | 4.38E-09 | 0.000 | 2.29 | 1.73E-48 | 5.92E-48 |
| *FXYD5* | 1.34 | 7.01E-10 | 1.75E-05 | 2.57 | 3.13E-51 | 1.46E-50 |
| *ARL14* | 1.72 | 2.94E-09 | 7.36E-05 | 2.15 | 1.24E-34 | 2.35E-34 |
| *CLRN3* | 1.21 | 1.21E-07 | 0.003 | 2.63 | 2.26E-53 | 1.81E-52 |
| *FXYD3* | 1.85 | 2.01E-08 | 0.001 | 1.99 | 6.46E-36 | 1.27E-35 |
| *CDH17* | 1.18 | 3.47E-07 | 0.009 | 2.65 | 4.64E-49 | 1.66E-48 |
| *KRT7* | 1.41 | 8.63E-07 | 0.022 | 2.42 | 6.59E-36 | 1.30E-35 |
| *PMEPA1* | 1.06 | 1.95E-08 | 0.000 | 2.77 | 1.85E-48 | 6.33E-48 |
| *ABHD17C* | 1.44 | 4.48E-08 | 0.001 | 2.38 | 4.74E-54 | 5.39E-53 |
| *TGM2* | 1.07 | 8.37E-07 | 0.021 | 2.75 | 1.13E-43 | 2.88E-43 |
| *COL6A3* | 1.77 | 7.98E-10 | 2.00E-05 | 2.04 | 1.72E-33 | 3.18E-33 |
| *EPHA2* | 1.02 | 1.10E-06 | 0.028 | 2.77 | 1.21E-48 | 4.18E-48 |
| *MIA* | 1.73 | 5.43E-09 | 0.000 | 2.06 | 1.12E-30 | 1.94E-30 |
| *MGLL* | 1.39 | 2.21E-10 | 5.52E-06 | 2.40 | 1.44E-54 | 2.20E-53 |
| *AMIGO2* | 1.37 | 5.49E-10 | 1.37E-05 | 2.40 | 2.33E-52 | 1.33E-51 |
| *ACTA2* | 1.20 | 1.08E-06 | 0.027 | 2.56 | 7.46E-41 | 1.69E-40 |
| *HPGD* | 1.34 | 2.76E-08 | 0.001 | 2.39 | 1.26E-49 | 4.76E-49 |
| *HEPH* | 1.34 | 9.07E-10 | 2.27E-05 | 2.37 | 1.25E-53 | 1.13E-52 |
| *SLC6A6* | 1.09 | 1.20E-06 | 0.030 | 2.61 | 1.50E-52 | 9.08E-52 |
| *LEMD1* | 1.77 | 2.77E-10 | 6.93E-06 | 1.93 | 8.91E-50 | 3.43E-49 |
| *ANXA1* | 1.35 | 1.69E-08 | 0.000 | 2.35 | 8.90E-45 | 2.38E-44 |
| *LGALS3* | 1.02 | 1.27E-08 | 0.000 | 2.66 | 8.38E-47 | 2.52E-46 |
| *OAS2* | 1.10 | 7.71E-07 | 0.019 | 2.57 | 4.24E-51 | 1.95E-50 |
| *KCNK1* | 1.24 | 3.14E-08 | 0.001 | 2.40 | 2.52E-52 | 1.44E-51 |
| *MBOAT2* | 1.66 | 9.21E-10 | 2.30E-05 | 1.98 | 3.97E-55 | 8.05E-54 |
| *MET* | 1.17 | 1.07E-06 | 0.027 | 2.45 | 2.22E-49 | 8.18E-49 |
| *MTMR11* | 1.55 | 5.93E-10 | 1.48E-05 | 2.06 | 4.54E-47 | 1.39E-46 |
| *CEP55* | 1.54 | 1.11E-11 | 2.78E-07 | 2.06 | 1.55E-54 | 2.33E-53 |
| *EPSTI1* | 1.42 | 1.11E-09 | 2.78E-05 | 2.18 | 4.31E-51 | 1.98E-50 |
| *LBH* | 1.05 | 3.54E-08 | 0.001 | 2.54 | 3.73E-51 | 1.73E-50 |
| *CYP2C18* | 1.41 | 1.49E-07 | 0.004 | 2.18 | 1.31E-46 | 3.91E-46 |
| *FHL2* | 1.45 | 5.16E-09 | 0.000 | 2.13 | 3.65E-48 | 1.22E-47 |
| *SLC16A4* | 1.45 | 9.67E-11 | 2.42E-06 | 2.12 | 1.60E-53 | 1.37E-52 |
| *FBN1* | 1.53 | 3.63E-08 | 0.001 | 2.05 | 1.21E-41 | 2.84E-41 |
| *ADAM9* | 1.30 | 1.81E-10 | 4.53E-06 | 2.27 | 5.75E-51 | 2.58E-50 |
| *SERPINH1* | 1.19 | 2.47E-08 | 0.001 | 2.38 | 4.43E-49 | 1.59E-48 |
| *SRPX2* | 1.18 | 2.83E-08 | 0.001 | 2.38 | 1.10E-47 | 3.51E-47 |
| *AFAP1-AS1* | 1.45 | 8.87E-07 | 0.022 | 2.11 | 4.14E-39 | 8.87E-39 |
| *LOX* | 1.40 | 2.62E-09 | 6.55E-05 | 2.15 | 1.98E-45 | 5.49E-45 |
| *RTP4* | 1.25 | 6.30E-08 | 0.002 | 2.30 | 2.62E-53 | 2.03E-52 |
| *ARNTL2* | 1.61 | 5.71E-10 | 1.43E-05 | 1.94 | 2.39E-54 | 3.24E-53 |
| *IGFL2* | 1.48 | 6.34E-09 | 0.000 | 2.04 | 3.23E-50 | 1.31E-49 |
| *ITGA3* | 1.56 | 5.02E-09 | 0.000 | 1.95 | 8.56E-49 | 3.00E-48 |
| *SCEL* | 1.46 | 9.32E-08 | 0.002 | 2.05 | 1.12E-42 | 2.73E-42 |
| *KRT6A* | 1.44 | 2.68E-09 | 6.71E-05 | 2.06 | 4.12E-15 | 5.51E-15 |
| *MFAP5* | 1.40 | 8.14E-09 | 0.000 | 2.10 | 1.21E-37 | 2.48E-37 |
| *FAP* | 2.31 | 6.76E-13 | 1.69E-08 | 1.18 | 1.26E-25 | 2.00E-25 |
| *SHISA2* | 1.52 | 4.04E-07 | 0.010 | 1.97 | 6.05E-56 | 1.89E-54 |
| *RARRES1* | 1.24 | 2.31E-07 | 0.006 | 2.22 | 2.66E-37 | 5.40E-37 |
| *MOXD1* | 1.25 | 1.64E-06 | 0.041 | 2.18 | 9.86E-48 | 3.16E-47 |
| *FAM83D* | 1.45 | 7.48E-10 | 1.87E-05 | 1.96 | 2.29E-54 | 3.13E-53 |
| *PLA2G7* | 1.40 | 2.05E-08 | 0.001 | 2.01 | 1.03E-52 | 6.54E-52 |
| *ID1* | 1.25 | 9.58E-09 | 0.000 | 2.15 | 3.63E-43 | 9.03E-43 |
| *NCF2* | 1.35 | 1.68E-09 | 4.19E-05 | 2.05 | 1.07E-42 | 2.62E-42 |
| *JUP* | 1.15 | 9.74E-08 | 0.002 | 2.24 | 3.49E-52 | 1.92E-51 |
| *PLS1* | 1.29 | 7.21E-07 | 0.018 | 2.10 | 9.86E-48 | 3.16E-47 |
| *GBP3* | 1.40 | 4.09E-10 | 1.02E-05 | 1.97 | 1.38E-39 | 3.00E-39 |
| *KRT6B* | 1.19 | 2.99E-07 | 0.007 | 2.18 | 3.06E-37 | 6.21E-37 |
| *ADAMTS12* | 1.30 | 1.01E-08 | 0.000 | 2.05 | 5.91E-48 | 1.93E-47 |
| *PXDN* | 1.40 | 6.50E-10 | 1.63E-05 | 1.94 | 2.33E-49 | 8.55E-49 |
| *BCL2A1* | 1.31 | 5.01E-07 | 0.013 | 2.03 | 2.98E-48 | 1.00E-47 |
| *STYK1* | 1.47 | 3.72E-09 | 9.30E-05 | 1.85 | 1.25E-53 | 1.13E-52 |
| *GPR137B* | 1.11 | 5.27E-07 | 0.013 | 2.20 | 3.87E-55 | 7.93E-54 |
| *NUSAP1* | 1.31 | 1.28E-09 | 3.21E-05 | 1.99 | 8.05E-51 | 3.52E-50 |
| *PTGS2* | 1.24 | 5.35E-07 | 0.013 | 2.06 | 2.54E-51 | 1.21E-50 |
| *GPR87* | 1.51 | 7.58E-11 | 1.90E-06 | 1.78 | 7.71E-45 | 2.07E-44 |
| *TGFBI* | 1.35 | 1.31E-09 | 3.29E-05 | 1.93 | 4.12E-38 | 8.56E-38 |
| *ECT2* | 1.48 | 5.49E-10 | 1.37E-05 | 1.81 | 3.22E-52 | 1.79E-51 |
| *EFNA5* | 1.15 | 1.95E-07 | 0.005 | 2.13 | 2.02E-55 | 4.75E-54 |
| *PKM* | 1.19 | 9.96E-11 | 2.49E-06 | 2.08 | 5.97E-55 | 1.12E-53 |
| *PPARG* | 1.27 | 8.74E-08 | 0.002 | 2.00 | 4.27E-47 | 1.31E-46 |
| *RHBDL2* | 1.26 | 1.80E-07 | 0.005 | 2.00 | 1.62E-51 | 7.91E-51 |
| *F5* | 1.34 | 9.03E-09 | 0.000 | 1.91 | 8.03E-38 | 1.65E-37 |
| *GPX8* | 1.33 | 2.18E-09 | 5.45E-05 | 1.93 | 8.19E-48 | 2.64E-47 |
| *RND3* | 1.04 | 6.68E-07 | 0.017 | 2.21 | 2.53E-54 | 3.39E-53 |
| *LEF1* | 1.45 | 4.60E-08 | 0.001 | 1.80 | 2.38E-49 | 8.74E-49 |
| *ALOX5AP* | 1.33 | 3.87E-09 | 9.70E-05 | 1.92 | 8.57E-39 | 1.82E-38 |
| *STEAP1* | 1.25 | 1.78E-06 | 0.045 | 1.99 | 4.16E-49 | 1.50E-48 |
| *MST1R* | 1.58 | 1.10E-07 | 0.003 | 1.66 | 4.74E-37 | 9.57E-37 |
| *PALLD* | 1.16 | 4.05E-08 | 0.001 | 2.07 | 7.89E-50 | 3.06E-49 |
| *IFI44* | 1.56 | 2.42E-12 | 6.04E-08 | 1.63 | 2.92E-37 | 5.93E-37 |
| *CENPW* | 1.03 | 9.92E-08 | 0.002 | 2.15 | 1.47E-55 | 3.72E-54 |
| *PTTG1* | 1.13 | 4.99E-09 | 0.000 | 2.06 | 1.29E-53 | 1.15E-52 |
| *CD109* | 1.24 | 1.81E-08 | 0.000 | 1.94 | 8.09E-53 | 5.29E-52 |
| *TNFRSF11B* | 1.19 | 9.81E-08 | 0.002 | 1.98 | 1.07E-44 | 2.84E-44 |
| *MKI67* | 1.12 | 1.21E-07 | 0.003 | 2.05 | 4.49E-53 | 3.19E-52 |
| *GBP2* | 1.38 | 7.37E-09 | 0.000 | 1.77 | 1.37E-44 | 3.64E-44 |
| *ENO2* | 1.33 | 1.26E-09 | 3.15E-05 | 1.81 | 3.14E-49 | 1.14E-48 |
| *OSBPL3* | 1.68 | 1.48E-12 | 3.71E-08 | 1.46 | 4.40E-45 | 1.19E-44 |
| *DDX60* | 1.43 | 3.28E-10 | 8.20E-06 | 1.71 | 1.20E-48 | 4.15E-48 |
| *ADAM12* | 1.18 | 2.58E-07 | 0.006 | 1.93 | 2.22E-49 | 8.18E-49 |
| *MELK* | 1.54 | 3.07E-10 | 7.67E-06 | 1.57 | 2.93E-50 | 1.19E-49 |
| *SPRR1B* | 1.14 | 9.05E-08 | 0.002 | 1.96 | 2.21E-17 | 3.05E-17 |
| *DLGAP5* | 1.51 | 1.24E-11 | 3.09E-07 | 1.58 | 3.13E-53 | 2.35E-52 |
| *CCNB1* | 1.33 | 2.55E-09 | 6.39E-05 | 1.74 | 8.61E-55 | 1.49E-53 |
| *PCDH7* | 1.21 | 1.78E-10 | 4.45E-06 | 1.86 | 1.49E-50 | 6.29E-50 |
| *IFI44L* | 1.79 | 8.18E-12 | 2.05E-07 | 1.26 | 2.42E-31 | 4.28E-31 |
| *WNT2* | 1.07 | 2.49E-08 | 0.001 | 1.98 | 1.36E-48 | 4.69E-48 |
| *BIRC3* | 1.03 | 1.74E-07 | 0.004 | 1.94 | 1.42E-39 | 3.09E-39 |
| *PSMB8* | 1.05 | 6.41E-09 | 0.000 | 1.92 | 1.39E-50 | 5.87E-50 |
| *EGLN3* | 1.47 | 3.04E-07 | 0.008 | 1.50 | 4.84E-29 | 8.15E-29 |
| *DACT1* | 1.17 | 5.86E-07 | 0.015 | 1.80 | 4.87E-48 | 1.61E-47 |
| *NCEH1* | 1.09 | 3.08E-08 | 0.001 | 1.87 | 1.73E-50 | 7.27E-50 |
| *PRRX1* | 1.27 | 7.26E-08 | 0.002 | 1.69 | 9.44E-39 | 2.00E-38 |
| *CTSB* | 1.06 | 1.06E-07 | 0.003 | 1.89 | 1.16E-47 | 3.70E-47 |
| *ITGB6* | 1.03 | 1.72E-06 | 0.043 | 1.91 | 1.80E-37 | 3.68E-37 |
| *PMAIP1* | 1.29 | 3.12E-07 | 0.008 | 1.65 | 2.10E-50 | 8.70E-50 |
| *XDH* | 1.15 | 1.53E-06 | 0.038 | 1.78 | 1.72E-49 | 6.42E-49 |
| *KIF11* | 1.28 | 1.22E-10 | 3.06E-06 | 1.65 | 3.17E-56 | 1.19E-54 |
| *NOX4* | 1.86 | 1.09E-10 | 2.72E-06 | 1.06 | 7.38E-40 | 1.62E-39 |
| *FBXO32* | 1.35 | 2.74E-08 | 0.001 | 1.55 | 2.27E-41 | 5.26E-41 |
| *RSAD2* | 1.48 | 1.99E-11 | 4.99E-07 | 1.43 | 1.46E-44 | 3.86E-44 |
| *EPYC* | 1.52 | 1.97E-10 | 4.92E-06 | 1.38 | 7.34E-49 | 2.59E-48 |
| *GIMAP2* | 1.14 | 1.46E-06 | 0.037 | 1.76 | 3.54E-50 | 1.43E-49 |
| *CDK1* | 1.03 | 1.99E-07 | 0.005 | 1.86 | 5.32E-52 | 2.82E-51 |
| *NDC80* | 1.45 | 1.94E-11 | 4.86E-07 | 1.44 | 4.98E-54 | 5.58E-53 |
| *FCER1G* | 1.03 | 9.29E-07 | 0.023 | 1.82 | 6.13E-37 | 1.23E-36 |
| *CD58* | 1.04 | 1.38E-07 | 0.003 | 1.81 | 5.44E-50 | 2.15E-49 |
| *PLEKHG1* | 1.07 | 2.56E-08 | 0.001 | 1.77 | 8.16E-54 | 8.18E-53 |
| *TMEM200A* | 1.34 | 9.17E-09 | 0.000 | 1.50 | 1.24E-47 | 3.97E-47 |
| *PSMB9* | 1.25 | 4.96E-10 | 1.24E-05 | 1.54 | 7.01E-42 | 1.66E-41 |
| *SQLE* | 1.03 | 1.25E-07 | 0.003 | 1.73 | 9.70E-49 | 3.38E-48 |
| *CDCA7* | 1.16 | 4.96E-09 | 0.000 | 1.59 | 5.06E-35 | 9.73E-35 |
| *MXD1* | 1.07 | 2.05E-08 | 0.001 | 1.68 | 3.89E-51 | 1.79E-50 |
| *OCIAD2* | 1.16 | 2.15E-08 | 0.001 | 1.59 | 1.64E-50 | 6.89E-50 |
| *IFI16* | 1.16 | 1.47E-06 | 0.037 | 1.58 | 1.60E-40 | 3.58E-40 |
| *LRRN1* | 1.30 | 1.83E-09 | 4.58E-05 | 1.43 | 6.19E-38 | 1.28E-37 |
| *ABRACL* | 1.01 | 3.63E-07 | 0.009 | 1.71 | 3.39E-47 | 1.05E-46 |
| *ITGBL1* | 1.37 | 6.25E-08 | 0.002 | 1.34 | 4.00E-26 | 6.39E-26 |
| *SLC26A9* | 1.34 | 1.81E-06 | 0.045 | 1.36 | 1.70E-16 | 2.32E-16 |
| *ANKRD22* | 1.42 | 6.48E-08 | 0.002 | 1.28 | 4.38E-22 | 6.55E-22 |
| *RHPN2* | 1.14 | 6.13E-08 | 0.002 | 1.55 | 2.29E-41 | 5.29E-41 |
| *NEK2* | 1.08 | 1.05E-08 | 0.000 | 1.61 | 5.66E-53 | 3.91E-52 |
| *KIF20A* | 1.12 | 5.37E-09 | 0.000 | 1.55 | 9.41E-54 | 9.09E-53 |
| *RGS1* | 1.15 | 5.71E-07 | 0.014 | 1.52 | 7.60E-25 | 1.19E-24 |
| *IL1R2* | 1.14 | 4.69E-08 | 0.001 | 1.51 | 3.18E-31 | 5.61E-31 |
| *GBP1* | 1.26 | 5.05E-08 | 0.001 | 1.39 | 5.39E-38 | 1.12E-37 |
| *RACGAP1* | 1.19 | 9.68E-09 | 0.000 | 1.46 | 1.24E-54 | 1.99E-53 |
| *SERPINB2* | 1.48 | 6.30E-09 | 0.000 | 1.17 | 9.00E-25 | 1.41E-24 |
| *NPR3* | 1.34 | 2.06E-09 | 5.16E-05 | 1.30 | 2.37E-45 | 6.55E-45 |
| *TMPRSS3* | 1.39 | 2.34E-07 | 0.006 | 1.24 | 8.02E-35 | 1.53E-34 |
| *SLC12A2* | 1.01 | 1.71E-08 | 0.000 | 1.62 | 7.11E-45 | 1.91E-44 |
| *IL18* | 1.21 | 5.93E-10 | 1.48E-05 | 1.41 | 3.66E-39 | 7.85E-39 |
| *ADAM28* | 1.19 | 5.94E-08 | 0.001 | 1.43 | 1.17E-34 | 2.23E-34 |
| *MX1* | 1.38 | 8.31E-11 | 2.08E-06 | 1.21 | 1.99E-28 | 3.32E-28 |
| *CENPF* | 1.23 | 5.73E-08 | 0.001 | 1.36 | 6.07E-47 | 1.85E-46 |
| *MCU* | 1.03 | 1.07E-07 | 0.003 | 1.55 | 1.90E-52 | 1.12E-51 |
| *MAP4K4* | 1.01 | 2.85E-07 | 0.007 | 1.56 | 3.46E-53 | 2.55E-52 |
| *PTPRR* | 1.44 | 7.34E-09 | 0.000 | 1.11 | 5.39E-50 | 2.13E-49 |
| *SPOCK1* | 1.21 | 1.25E-09 | 3.12E-05 | 1.33 | 5.86E-30 | 1.01E-29 |
| *ARPC1B* | 1.10 | 6.52E-09 | 0.000 | 1.44 | 3.99E-45 | 1.09E-44 |
| *RAI14* | 1.17 | 8.61E-09 | 0.000 | 1.36 | 1.48E-48 | 5.09E-48 |
| *CALD1* | 1.05 | 1.00E-07 | 0.003 | 1.48 | 8.74E-35 | 1.67E-34 |
| *SERPINB3* | 1.38 | 1.98E-09 | 4.96E-05 | 1.14 | 1.14E-16 | 1.56E-16 |
| *INPP4B* | 1.11 | 9.36E-09 | 0.000 | 1.39 | 2.53E-47 | 7.90E-47 |
| *SAMD9L* | 1.08 | 3.96E-09 | 9.92E-05 | 1.41 | 3.14E-44 | 8.19E-44 |
| *NDNF* | 1.12 | 1.79E-07 | 0.004 | 1.35 | 3.22E-48 | 1.08E-47 |
| *ENTPD1* | 1.24 | 1.69E-07 | 0.004 | 1.22 | 1.36E-52 | 8.31E-52 |
| *PBK* | 1.10 | 3.76E-08 | 0.001 | 1.35 | 1.74E-53 | 1.47E-52 |
| *C5orf46* | 1.09 | 1.30E-07 | 0.003 | 1.35 | 3.81E-37 | 7.71E-37 |
| *FRMD6* | 1.17 | 7.01E-07 | 0.018 | 1.26 | 5.39E-38 | 1.12E-37 |
| *TREM1* | 1.35 | 4.21E-07 | 0.011 | 1.07 | 1.97E-33 | 3.64E-33 |
| *NUAK1* | 1.02 | 5.09E-08 | 0.001 | 1.38 | 3.78E-50 | 1.52E-49 |
| *CDKN3* | 1.03 | 1.54E-09 | 3.85E-05 | 1.37 | 4.09E-50 | 1.64E-49 |
| *BUB1B* | 1.28 | 3.42E-09 | 8.56E-05 | 1.10 | 5.88E-49 | 2.09E-48 |
| *C1GALT1* | 1.04 | 6.92E-09 | 0.000 | 1.32 | 4.88E-53 | 3.42E-52 |
| *EGFL6* | 1.22 | 4.64E-08 | 0.001 | 1.13 | 2.25E-53 | 1.80E-52 |
| *DTL* | 1.22 | 1.18E-10 | 2.94E-06 | 1.13 | 7.81E-53 | 5.14E-52 |
| *TNFSF4* | 1.22 | 2.22E-09 | 5.56E-05 | 1.12 | 1.61E-46 | 4.76E-46 |
| *ETV1* | 1.04 | 9.97E-07 | 0.025 | 1.29 | 7.00E-52 | 3.63E-51 |
| *EPHA4* | 1.06 | 2.30E-07 | 0.006 | 1.27 | 5.14E-48 | 1.69E-47 |
| *TMEM154* | 1.27 | 3.43E-08 | 0.001 | 1.04 | 7.04E-49 | 2.48E-48 |
| *NUF2* | 1.18 | 5.78E-09 | 0.000 | 1.13 | 7.23E-52 | 3.74E-51 |
| *HMMR* | 1.07 | 5.37E-09 | 0.000 | 1.24 | 4.38E-52 | 2.36E-51 |
| *HSD17B6* | 1.30 | 1.25E-11 | 3.13E-07 | 1.01 | 4.39E-45 | 1.19E-44 |
| *FGD6* | 1.18 | 8.70E-09 | 0.000 | 1.12 | 2.02E-43 | 5.07E-43 |
| *MPZL2* | 1.07 | 4.20E-08 | 0.001 | 1.22 | 1.50E-35 | 2.92E-35 |
| *IFIT1* | 1.07 | 7.39E-08 | 0.002 | 1.19 | 3.77E-28 | 6.24E-28 |
| *DSG3* | 1.10 | 1.34E-06 | 0.034 | 1.14 | 6.86E-24 | 1.06E-23 |
| *ZNF521* | 1.19 | 1.99E-06 | 0.050 | 1.05 | 1.29E-41 | 3.00E-41 |
| *RAD51AP1* | 1.20 | 3.50E-10 | 8.77E-06 | 1.03 | 1.30E-47 | 4.15E-47 |
| *HTR2B* | 1.14 | 3.12E-09 | 7.82E-05 | 1.08 | 2.57E-47 | 8.01E-47 |
| *MYO1E* | 1.10 | 7.06E-10 | 1.77E-05 | 1.04 | 8.09E-40 | 1.77E-39 |
| *SHCBP1* | 1.06 | 3.07E-09 | 7.68E-05 | 1.04 | 7.04E-49 | 2.48E-48 |
| *NCAPG* | 1.01 | 1.14E-07 | 0.003 | 1.07 | 8.97E-49 | 3.14E-48 |
| *NTRK2* | -1.06 | 4.81E-07 | 0.012 | -1.06 | 9.65E-30 | 1.65E-29 |
| *MT1E* | -1.01 | 3.77E-10 | 9.44E-06 | -1.13 | 1.39E-18 | 1.97E-18 |
| *DIRAS3* | -1.18 | 1.57E-08 | 0.000 | -1.02 | 2.16E-30 | 3.73E-30 |
| *LIFR* | -1.23 | 8.18E-11 | 2.05E-06 | -1.04 | 1.61E-34 | 3.05E-34 |
| *PHYHIPL* | -1.03 | 6.57E-09 | 0.000 | -1.26 | 2.98E-42 | 7.13E-42 |
| *SLC25A15* | -1.15 | 5.08E-09 | 0.000 | -1.16 | 5.25E-38 | 1.09E-37 |
| *TIFA* | -1.02 | 2.28E-09 | 5.71E-05 | -1.31 | 3.28E-47 | 1.02E-46 |
| *DLK1* | -1.11 | 6.37E-08 | 0.002 | -1.29 | 3.60E-22 | 5.38E-22 |
| *JADE1* | -1.10 | 1.57E-09 | 3.92E-05 | -1.31 | 1.20E-46 | 3.59E-46 |
| *CCDC69* | -1.27 | 1.13E-10 | 2.83E-06 | -1.16 | 3.31E-30 | 5.71E-30 |
| *VEPH1* | -1.08 | 3.99E-08 | 0.001 | -1.36 | 7.23E-48 | 2.35E-47 |
| *PBLD* | -1.21 | 3.88E-11 | 9.70E-07 | -1.26 | 1.46E-34 | 2.77E-34 |
| *CTH* | -1.24 | 1.75E-10 | 4.38E-06 | -1.22 | 4.48E-42 | 1.06E-41 |
| *MPV17L* | -1.09 | 8.31E-10 | 2.08E-05 | -1.38 | 3.23E-43 | 8.05E-43 |
| *F8* | -1.52 | 1.92E-14 | 4.80E-10 | -1.08 | 5.85E-40 | 1.29E-39 |
| *BCAT1* | -1.12 | 1.91E-08 | 0.000 | -1.51 | 1.91E-36 | 3.79E-36 |
| *PDZK1* | -1.47 | 2.29E-10 | 5.73E-06 | -1.18 | 9.27E-24 | 1.42E-23 |
| *KCNK3* | -1.09 | 3.48E-07 | 0.009 | -1.59 | 4.12E-30 | 7.10E-30 |
| *SLC39A8* | -1.06 | 8.00E-09 | 0.000 | -1.64 | 1.85E-43 | 4.65E-43 |
| *CD36* | -1.00 | 9.05E-07 | 0.023 | -1.71 | 7.60E-38 | 1.57E-37 |
| *SERPINA5* | -1.42 | 4.58E-10 | 1.15E-05 | -1.29 | 6.13E-15 | 8.16E-15 |
| *SLC7A2* | -1.32 | 1.96E-08 | 0.000 | -1.43 | 2.08E-30 | 3.60E-30 |
| *GRPR* | -1.25 | 8.51E-08 | 0.002 | -1.52 | 3.14E-44 | 8.19E-44 |
| *GAS2* | -1.56 | 3.01E-11 | 7.53E-07 | -1.23 | 5.59E-45 | 1.51E-44 |
| *CFTR* | -1.32 | 7.47E-08 | 0.002 | -1.49 | 3.48E-17 | 4.80E-17 |
| *SLC41A1* | -1.24 | 4.67E-12 | 1.17E-07 | -1.57 | 4.91E-49 | 1.75E-48 |
| *SFRP5* | -1.23 | 1.77E-09 | 4.42E-05 | -1.58 | 2.36E-11 | 2.97E-11 |
| *ECHDC3* | -1.19 | 6.22E-10 | 1.56E-05 | -1.63 | 4.70E-46 | 1.35E-45 |
| *ALDH1L2* | -1.02 | 1.96E-06 | 0.049 | -1.81 | 1.18E-46 | 3.54E-46 |
| *LPAR3* | -1.25 | 2.82E-09 | 7.05E-05 | -1.58 | 2.36E-49 | 8.68E-49 |
| *CLU* | -1.03 | 1.01E-07 | 0.003 | -1.83 | 2.27E-28 | 3.76E-28 |
| *AMBP* | -1.18 | 7.66E-09 | 0.000 | -1.69 | 6.36E-15 | 8.48E-15 |
| *CHRM3* | -1.24 | 1.62E-10 | 4.06E-06 | -1.67 | 1.64E-49 | 6.14E-49 |
| *PRDX4* | -1.16 | 3.03E-09 | 7.58E-05 | -1.76 | 6.11E-43 | 1.51E-42 |
| *BTG2* | -1.50 | 7.56E-14 | 1.89E-09 | -1.44 | 2.35E-31 | 4.14E-31 |
| *C6* | -1.93 | 7.22E-13 | 1.81E-08 | -1.03 | 1.14E-09 | 1.41E-09 |
| *NPY1R* | -1.37 | 4.36E-13 | 1.09E-08 | -1.59 | 8.45E-39 | 1.79E-38 |
| *MT1M* | -1.67 | 4.15E-12 | 1.04E-07 | -1.33 | 4.99E-18 | 6.98E-18 |
| *SLC1A2* | -1.33 | 7.36E-10 | 1.84E-05 | -1.70 | 4.50E-49 | 1.61E-48 |
| *SLC17A4* | -1.21 | 5.50E-07 | 0.014 | -1.87 | 3.47E-42 | 8.28E-42 |
| *IGFBP2* | -1.16 | 2.24E-10 | 5.61E-06 | -1.92 | 4.04E-35 | 7.79E-35 |
| *GCG* | -1.97 | 1.96E-11 | 4.90E-07 | -1.13 | 0.038 | 0.039 |
| *GCAT* | -1.10 | 9.87E-09 | 0.000 | -2.00 | 1.53E-42 | 3.69E-42 |
| *TMEM97* | -1.56 | 5.14E-12 | 1.29E-07 | -1.55 | 5.59E-35 | 1.07E-34 |
| *VLDLR* | -1.23 | 8.93E-11 | 2.23E-06 | -1.88 | 7.92E-51 | 3.47E-50 |
| *ADH1B* | -1.29 | 3.55E-08 | 0.001 | -1.85 | 5.89E-28 | 9.71E-28 |
| *AKAP7* | -1.27 | 2.59E-09 | 6.47E-05 | -1.92 | 1.43E-43 | 3.61E-43 |
| *DPP10* | -1.65 | 1.99E-10 | 4.98E-06 | -1.53 | 1.91E-52 | 1.12E-51 |
| *MT1F* | -1.43 | 1.10E-12 | 2.76E-08 | -1.76 | 6.47E-37 | 1.30E-36 |
| *SERPINI1* | -1.46 | 1.57E-11 | 3.92E-07 | -1.77 | 9.45E-45 | 2.52E-44 |
| *RAB26* | -1.10 | 5.84E-09 | 0.000 | -2.14 | 1.16E-38 | 2.46E-38 |
| *GSTA1* | -1.50 | 2.69E-10 | 6.73E-06 | -1.74 | 7.38E-22 | 1.10E-21 |
| *TEX11* | -1.49 | 1.04E-07 | 0.003 | -1.76 | 4.88E-42 | 1.16E-41 |
| *PDK4* | -1.76 | 6.11E-13 | 1.53E-08 | -1.49 | 5.54E-19 | 7.88E-19 |
| *CXCL12* | -1.28 | 2.22E-08 | 0.001 | -1.98 | 5.48E-35 | 1.05E-34 |
| *CTNND2* | -1.32 | 1.75E-10 | 4.38E-06 | -1.95 | 5.88E-49 | 2.09E-48 |
| *SLC4A4* | -1.81 | 1.76E-13 | 4.41E-09 | -1.51 | 2.65E-18 | 3.72E-18 |
| *ECI2* | -1.34 | 1.59E-10 | 3.98E-06 | -1.98 | 1.85E-50 | 7.72E-50 |
| *PHGDH* | -1.16 | 3.25E-10 | 8.14E-06 | -2.20 | 1.92E-47 | 6.03E-47 |
| *ZBTB16* | -1.51 | 6.22E-10 | 1.56E-05 | -1.88 | 5.90E-43 | 1.45E-42 |
| *LGALS2* | -2.00 | 5.96E-15 | 1.49E-10 | -1.40 | 1.58E-13 | 2.06E-13 |
| *CCDC110* | -1.54 | 5.71E-11 | 1.43E-06 | -1.86 | 5.62E-51 | 2.52E-50 |
| *MCOLN3* | -1.10 | 2.72E-09 | 6.81E-05 | -2.32 | 8.45E-51 | 3.68E-50 |
| *ABAT* | -1.47 | 4.74E-10 | 1.19E-05 | -1.98 | 9.52E-40 | 2.08E-39 |
| *RNF186* | -1.14 | 6.24E-09 | 0.000 | -2.33 | 7.96E-41 | 1.80E-40 |
| *GMNN* | -1.26 | 1.93E-09 | 4.84E-05 | -2.24 | 6.38E-51 | 2.83E-50 |
| *TDH* | -1.14 | 4.20E-09 | 0.000 | -2.39 | 6.63E-53 | 4.48E-52 |
| *SLC3A1* | -1.48 | 2.12E-10 | 5.30E-06 | -2.11 | 1.77E-30 | 3.07E-30 |
| *NRCAM* | -1.56 | 1.33E-10 | 3.32E-06 | -2.04 | 2.39E-41 | 5.52E-41 |
| *SIDT2* | -1.22 | 3.60E-11 | 9.00E-07 | -2.41 | 6.81E-54 | 7.14E-53 |
| *SEL1L* | -1.59 | 4.89E-12 | 1.22E-07 | -2.05 | 1.29E-43 | 3.29E-43 |
| *LMO3* | -1.17 | 4.83E-08 | 0.001 | -2.49 | 7.84E-52 | 4.03E-51 |
| *TCEA3* | -1.21 | 7.16E-11 | 1.79E-06 | -2.47 | 6.11E-49 | 2.17E-48 |
| *PAK3* | -1.59 | 2.81E-12 | 7.02E-08 | -2.09 | 8.26E-45 | 2.22E-44 |
| *CA4* | -1.16 | 2.08E-08 | 0.001 | -2.53 | 4.23E-48 | 1.40E-47 |
| *COCH* | -1.10 | 2.80E-09 | 7.00E-05 | -2.62 | 4.20E-48 | 1.39E-47 |
| *C5* | -1.75 | 5.14E-12 | 1.29E-07 | -2.00 | 2.20E-48 | 7.46E-48 |
| *TRHDE* | -2.31 | 1.37E-14 | 3.42E-10 | -1.46 | 6.39E-48 | 2.08E-47 |
| *LINC00339* | -1.67 | 3.42E-11 | 8.56E-07 | -2.13 | 1.10E-48 | 3.82E-48 |
| *RGN* | -1.52 | 9.94E-11 | 2.49E-06 | -2.28 | 3.60E-48 | 1.20E-47 |
| *SLC16A10* | -1.81 | 4.18E-13 | 1.05E-08 | -2.01 | 3.34E-49 | 1.21E-48 |
| *EPHX2* | -1.44 | 2.93E-11 | 7.33E-07 | -2.39 | 1.47E-49 | 5.53E-49 |
| *GRB14* | -1.43 | 4.43E-10 | 1.11E-05 | -2.41 | 1.68E-52 | 1.00E-51 |
| *MAT1A* | -1.20 | 1.19E-11 | 2.97E-07 | -2.68 | 4.95E-49 | 1.77E-48 |
| *KCNJ16* | -1.92 | 4.69E-11 | 1.17E-06 | -1.97 | 6.09E-35 | 1.17E-34 |
| *GAMT* | -1.22 | 9.20E-11 | 2.30E-06 | -2.67 | 4.23E-48 | 1.40E-47 |
| *PSAT1* | -1.93 | 6.52E-12 | 1.63E-07 | -2.11 | 1.07E-39 | 2.32E-39 |
| *ADHFE1* | -1.07 | 3.53E-09 | 8.84E-05 | -2.99 | 1.82E-54 | 2.65E-53 |
| *ACSM3* | -1.17 | 7.16E-11 | 1.79E-06 | -2.92 | 2.49E-53 | 1.96E-52 |
| *GPT2* | -1.34 | 9.12E-11 | 2.28E-06 | -2.77 | 8.32E-47 | 2.51E-46 |
| *IL22RA1* | -1.59 | 1.70E-11 | 4.25E-07 | -2.54 | 4.84E-44 | 1.25E-43 |
| *MT1H* | -1.29 | 2.26E-12 | 5.65E-08 | -2.85 | 5.08E-36 | 1.00E-35 |
| *KIRREL2* | -1.04 | 6.86E-08 | 0.002 | -3.19 | 3.24E-49 | 1.18E-48 |
| *BACE1* | -1.86 | 1.06E-13 | 2.65E-09 | -2.37 | 2.94E-49 | 1.07E-48 |
| *IAPP* | -3.00 | 1.26E-17 | 3.15E-13 | -1.26 | 7.31E-11 | 9.16E-11 |
| *MT1X* | -1.14 | 4.24E-11 | 1.06E-06 | -3.13 | 3.67E-49 | 1.33E-48 |
| *SEC11C* | -1.14 | 6.85E-09 | 0.000 | -3.14 | 5.31E-51 | 2.39E-50 |
| *F11* | -1.54 | 5.60E-07 | 0.014 | -2.74 | 2.66E-50 | 1.09E-49 |
| *NUCB2* | -1.35 | 2.94E-10 | 7.36E-06 | -3.02 | 5.10E-50 | 2.02E-49 |
| *NR5A2* | -2.12 | 3.54E-14 | 8.86E-10 | -2.27 | 1.65E-48 | 5.66E-48 |
| *FAM3B* | -1.56 | 1.56E-10 | 3.90E-06 | -2.85 | 3.29E-49 | 1.19E-48 |
| *PDCD4* | -1.07 | 3.95E-10 | 9.87E-06 | -3.35 | 1.53E-54 | 2.32E-53 |
| *EPB41L4B* | -1.32 | 8.48E-12 | 2.12E-07 | -3.15 | 1.60E-50 | 6.73E-50 |
| *RBP1* | -1.20 | 5.35E-09 | 0.000 | -3.30 | 1.98E-53 | 1.63E-52 |
| *KIAA1324* | -2.15 | 5.62E-14 | 1.41E-09 | -2.36 | 2.17E-35 | 4.21E-35 |
| *SLC30A2* | -1.20 | 6.94E-09 | 0.000 | -3.31 | 2.23E-45 | 6.17E-45 |
| *NRG4* | -1.50 | 4.21E-09 | 0.000 | -3.13 | 1.85E-52 | 1.09E-51 |
| *SYBU* | -1.67 | 2.16E-10 | 5.40E-06 | -2.98 | 3.90E-50 | 1.57E-49 |
| *MT1G* | -1.53 | 1.75E-11 | 4.37E-07 | -3.13 | 4.21E-35 | 8.10E-35 |
| *SLC38A5* | -1.19 | 6.00E-08 | 0.002 | -3.49 | 2.04E-50 | 8.50E-50 |
| *IMPA2* | -1.40 | 1.79E-10 | 4.47E-06 | -3.32 | 1.13E-51 | 5.70E-51 |
| *FKBP11* | -1.15 | 6.15E-11 | 1.54E-06 | -3.60 | 1.62E-51 | 7.91E-51 |
| *DPEP1* | -1.50 | 1.64E-10 | 4.10E-06 | -3.39 | 1.02E-47 | 3.26E-47 |
| *ANPEP* | -1.73 | 2.41E-10 | 6.04E-06 | -3.23 | 6.19E-41 | 1.41E-40 |
| *AZGP1* | -1.79 | 6.25E-11 | 1.56E-06 | -3.18 | 4.28E-46 | 1.23E-45 |
| *GUCA1C* | -1.84 | 7.86E-11 | 1.97E-06 | -3.13 | 8.10E-51 | 3.54E-50 |
| *PAIP2B* | -2.29 | 3.51E-15 | 8.77E-11 | -2.69 | 4.91E-48 | 1.62E-47 |
| *MKNK1* | -1.01 | 1.82E-08 | 0.000 | -4.04 | 2.02E-55 | 4.75E-54 |
| *P2RX1* | -1.67 | 9.63E-12 | 2.41E-07 | -3.39 | 7.92E-51 | 3.47E-50 |
| *REG3A* | -1.68 | 2.19E-10 | 5.47E-06 | -3.42 | 8.29E-12 | 1.05E-11 |
| *GLS2* | -1.02 | 2.07E-08 | 0.001 | -4.09 | 4.96E-53 | 3.47E-52 |
| *SLC43A1* | -1.82 | 2.24E-13 | 5.60E-09 | -3.29 | 4.08E-50 | 1.64E-49 |
| *GPHA2* | -1.88 | 1.14E-14 | 2.84E-10 | -3.37 | 1.54E-43 | 3.89E-43 |
| *ACADL* | -1.89 | 9.04E-12 | 2.26E-07 | -3.49 | 4.98E-54 | 5.58E-53 |
| *AOX1* | -2.32 | 3.63E-16 | 9.08E-12 | -3.07 | 1.61E-49 | 6.00E-49 |
| *HPN* | -1.22 | 1.12E-08 | 0.000 | -4.18 | 2.60E-51 | 1.23E-50 |
| *CYB5A* | -1.16 | 3.38E-10 | 8.47E-06 | -4.26 | 4.08E-54 | 4.82E-53 |
| *TPST2* | -1.63 | 1.60E-11 | 4.02E-07 | -3.89 | 3.88E-51 | 1.79E-50 |
| *GATM* | -1.52 | 1.23E-11 | 3.08E-07 | -4.01 | 5.85E-48 | 1.91E-47 |
| *SPX* | -2.11 | 2.87E-11 | 7.18E-07 | -3.59 | 1.34E-54 | 2.10E-53 |
| *HOMER2* | -1.85 | 1.37E-13 | 3.43E-09 | -3.99 | 2.41E-53 | 1.91E-52 |
| *EGF* | -2.52 | 1.98E-14 | 4.97E-10 | -3.44 | 2.84E-51 | 1.34E-50 |
| *BNIP3* | -1.93 | 7.25E-15 | 1.81E-10 | -4.04 | 8.22E-53 | 5.35E-52 |
| *PM20D1* | -2.27 | 2.52E-13 | 6.30E-09 | -3.80 | 7.41E-47 | 2.24E-46 |
| *FXYD2* | -1.25 | 5.66E-09 | 0.000 | -4.87 | 8.22E-54 | 8.23E-53 |
| *TMEM52* | -1.58 | 1.45E-06 | 0.036 | -4.89 | 2.58E-51 | 1.22E-50 |
| *SLC39A5* | -1.62 | 1.35E-11 | 3.37E-07 | -4.86 | 1.79E-50 | 7.48E-50 |
| *TMED6* | -3.02 | 1.19E-16 | 2.97E-12 | -3.74 | 1.43E-46 | 4.26E-46 |
| *PRSS3* | -1.93 | 3.33E-13 | 8.34E-09 | -4.86 | 2.34E-47 | 7.32E-47 |
| *REG3G* | -1.62 | 4.28E-09 | 0.000 | -5.29 | 1.99E-36 | 3.96E-36 |
| *CBS* | -1.41 | 1.17E-10 | 2.92E-06 | -5.67 | 3.18E-54 | 4.07E-53 |
| *ERP27* | -3.06 | 1.30E-19 | 3.26E-15 | -4.13 | 5.41E-44 | 1.39E-43 |
| *PRSS2* | -1.63 | 7.98E-10 | 2.00E-05 | -6.13 | 1.37E-41 | 3.20E-41 |
| *REG1A* | -1.95 | 2.46E-12 | 6.15E-08 | -5.85 | 1.94E-41 | 4.49E-41 |
| *PDIA2* | -2.09 | 1.76E-13 | 4.41E-09 | -5.83 | 1.10E-46 | 3.30E-46 |
| *AQP8* | -2.73 | 1.50E-16 | 3.75E-12 | -5.20 | 3.61E-45 | 9.86E-45 |
| *FGL1* | -2.59 | 3.94E-15 | 9.86E-11 | -5.36 | 4.01E-48 | 1.33E-47 |
| *GNMT* | -2.60 | 5.62E-14 | 1.41E-09 | -5.36 | 2.00E-50 | 8.31E-50 |
| *KLK1* | -2.73 | 2.14E-16 | 5.36E-12 | -5.96 | 6.48E-49 | 2.30E-48 |
| *ALB* | -3.20 | 1.19E-16 | 2.97E-12 | -5.53 | 8.17E-51 | 3.57E-50 |
| *RBPJL* | -2.23 | 4.12E-14 | 1.03E-09 | -6.67 | 3.82E-53 | 2.79E-52 |
| *CELA2A* | -1.05 | 7.88E-07 | 0.020 | -7.88 | 1.21E-42 | 2.94E-42 |
| *PNLIP* | -2.40 | 9.04E-13 | 2.26E-08 | -6.75 | 8.78E-26 | 1.40E-25 |
| *SERPINI2* | -3.50 | 5.23E-20 | 1.31E-15 | -5.70 | 4.77E-49 | 1.70E-48 |
| *CELP* | -2.62 | 2.55E-14 | 6.38E-10 | -6.61 | 1.13E-54 | 1.84E-53 |
| *PNLIPRP2* | -3.13 | 1.14E-17 | 2.86E-13 | -6.14 | 4.50E-38 | 9.34E-38 |
| *CELA2B* | -3.15 | 1.56E-19 | 3.91E-15 | -6.30 | 1.21E-41 | 2.83E-41 |
| *CUZD1* | -3.11 | 6.36E-17 | 1.59E-12 | -6.37 | 2.66E-47 | 8.31E-47 |
| *CPA2* | -2.96 | 6.14E-15 | 1.54E-10 | -6.76 | 8.88E-41 | 2.01E-40 |
| *CPB1* | -2.21 | 4.22E-12 | 1.06E-07 | -7.67 | 7.58E-45 | 2.04E-44 |
| *PLA2G1B* | -2.72 | 3.70E-14 | 9.27E-10 | -7.44 | 1.55E-42 | 3.75E-42 |
| *SYCN* | -3.12 | 7.39E-17 | 1.85E-12 | -7.04 | 2.57E-41 | 5.93E-41 |
| *REG1B* | -2.57 | 1.66E-13 | 4.15E-09 | -7.66 | 2.97E-46 | 8.67E-46 |
| *CELA3A* | -2.69 | 1.46E-13 | 3.65E-09 | -7.70 | 3.08E-44 | 8.02E-44 |
| *CEL* | -3.05 | 1.28E-17 | 3.19E-13 | -7.55 | 2.59E-46 | 7.56E-46 |
| *CTRC* | -3.10 | 3.09E-17 | 7.74E-13 | -7.64 | 4.50E-49 | 1.61E-48 |
| *GP2* | -2.96 | 9.89E-17 | 2.47E-12 | -7.82 | 4.65E-51 | 2.12E-50 |
| *CELA3B* | -2.64 | 4.87E-15 | 1.22E-10 | -8.17 | 1.03E-48 | 3.59E-48 |
| *CPA1* | -2.76 | 1.92E-14 | 4.80E-10 | -8.11 | 3.99E-44 | 1.04E-43 |
| *CTRB2* | -2.50 | 3.27E-14 | 8.18E-10 | -8.59 | 8.64E-54 | 8.53E-53 |
| *PNLIPRP1* | -3.30 | 6.69E-19 | 1.67E-14 | -7.85 | 1.14E-53 | 1.05E-52 |
| *CLPS* | -2.98 | 5.04E-17 | 1.26E-12 | -8.29 | 4.75E-45 | 1.29E-44 |
| *CTRL* | -3.37 | 2.22E-19 | 5.55E-15 | -8.38 | 8.32E-55 | 1.45E-53 |
